# Identification of a T cell gene expression clock obtained by exploiting a MZ twin design

**DOI:** 10.1038/s41598-017-05694-2

**Published:** 2017-07-20

**Authors:** Daniel Remondini, Nathan Intrator, Claudia Sala, Michela Pierini, Paolo Garagnani, Isabella Zironi, Claudio Franceschi, Stefano Salvioli, Gastone Castellani

**Affiliations:** 1 Department of Physics and Astronomy, University of Bologna, Bologna, 40126 Italy; 2 Interdepartmental Center “L. Galvani”, University of Bologna, Bologna, 40126 Italy; 3 Department of Computer Science, Exact Sciences Faculty, Tel Aviv University, Tel Aviv, Israel; 4 Department of Experimental, Diagnostic and Specialty Medicine, University of Bologna, Bologna, 40138 Italy; 5 IRCCS, Institute of Neurological Sciences of Bologna, Bologna, 40124 Italy; 6 Bone Regeneration Laboratory, Research Institute Codivilla-Putti, Rizzoli Orthopaedic Institute, Via di Barbiano 1/10, 40136 Bologna, Italy

## Abstract

Many studies investigated age-related changes in gene expression of different tissues, with scarce agreement due to the high number of affecting factors. Similarly, no consensus has been reached on which genes change expression as a function of age and not because of environment. In this study we analysed gene expression of T lymphocytes from 27 healthy monozygotic twin couples, with ages ranging over whole adult lifespan (22 to 98 years). This unique experimental design allowed us to identify genes involved in normative aging, which expression changes independently from environmental factors. We obtained a transcriptomic signature with 125 genes, from which chronological age can be estimated. This signature has been tested in two datasets of same cell type hybridized over two different platforms, showing a significantly better performance compared to random signatures. Moreover, the same signature was applied on a dataset from a different cell type (human muscle). A lower performance was obtained, indicating the possibility that the signature is T cell-specific. As a whole our results suggest that this approach can be useful to identify age-modulated genes.

## Introduction

Aging is a complex phenomenon characterised by decreased fitness and increased risk of diseases, disability and death. All these features are sustained by changes in gene expression, as a response of the cells to the environmental stimuli. Whether this response is programmed and stereotyped or totally random has been (and still is) a puzzling question for gerontologists. This question stems from the old theoretical dichotomy which has dominated the field of aging studies, that can be summarized in two conflicting positions: “aging is programmed” *vs* “aging is a random process”^1–3^. The fact that no gerontogenes (that is, genes whose expression actively induces aging of the organism without any other apparent benefit) have been found so far does not exclude that other (possibly epigenetic) types of control exist, so the question is still open. A third possibility also exists, that, according to the conceptualization of Blagosklonny and Hall^4^, aging is *quasi-programmed*, and should be interpreted as a continuation of developmental programs which, in the post-reproductive period of life, loose their strict and finely tuned modulation. A possible useful model to understand, at least in part, the presence of genetic (or epigenetic) control over age-related gene expression changes is that of twins^5^. Indeed, monozygotic (MZ) twins share the same genome and they can be therefore considered a powerful model to identify genes whose expression is independent from environmental perturbations, with the further possibility to cross-validate the data obtained in a member of the pair with those obtained in the other. Therefore, as MZ twins grow old, it should be possible to observe whether some of their genes change expression accordingly, indicating the presence of some kind of genetic control over this phenomenon, or rather if changes are totally private (not shared by the two members of the twin couple). Until today, a plethora of studies analyzed gene expression time series in different tissues (including brain areas, adipose tissue and skeletal muscle) from subjects of different age^6–14^, but not in twins. On the other side, gene expression studies on twin pairs have been performed so far in limited number of old subjects^15^, or in case-control studies^16, 17^. In some cases, a set of genes able to discriminate between young and old subjects has been identified^10, 18^ and a “transcriptomic signature of age” has been proposed, as in the case of the recent meta-analysis by Peters *et al*.^19^. However, inter-individual gene expression variability is a major factor that cannot be always overcome by a high number of studied subjects, especially when subjects of different ethnicity are mixed together. Moreover, in most cases, the observed changes can be related to the health status of the subjects, and not to age in itself. Due to the intrinsic difficulties in obtaining a general consensus on this topic, some researchers have turned their attention from gene expression to more stable aspects of DNA such as methylation, and developed signatures of age based on changes in CpGs methylation^20^, ^21^.

In this study, we aimed at analyzing gene expression in T cells from 27 couples of MZ twins from 20 to 98 years of age, in order to understand whether gene expression changes are conserved through genetically identical subjects and to identify which genes/pathways resulted more altered with age. The rationale of this analysis is to characterize the information on genetic variability embedded in a whole-genome expression time series of healthy MZ twin couples, in order to extract a low-dimensional gene signature for chronological age regression (“age signature”) independently from the effect of the environment. To find these genes, we used a measure able to characterize gene variability between twin pairs. With this measure, genes that have a low score are very similar in their expression between the twins, and are therefore likely to vary significantly with age with a high signal-to-noise ratio. From our characterization of gene variability over time on the MZ twin couples, we can suppose that these genes are less affected by environmental causes that produce a divergence in expression profile during aging. In order to test the goodness of the chosen signature, we compared its regression performance with a large set of randomly chosen signatures of the same dimensionality, providing a ranking of our signature against a null model. Moreover, in order to check whether the signature has a general relevance, not dependent on the used feature extraction methods, we tested the signature in different datasets obtained from cells of the same type hybridized on different microarray platforms, or from a different cell type. The results propose for a signature which is specific for the cell type considered.

## Results

### Global variability over time

**Figure 1:**
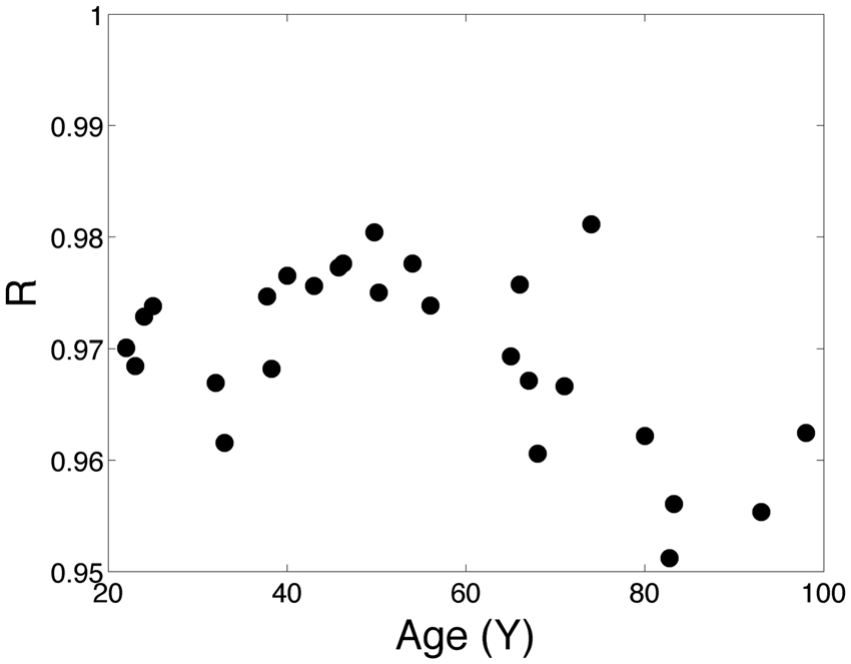
Plot of Pearson’s correlation coefficient R between MZ couples whole-genome expression profiles as a function of age.

To characterize the whole-genome behaviour over time, we considered Pearson’s correlation coefficient *R* of the whole array expression profile (comprising about 55,000 probes) for each twin couple, as shown in Fig. 1. The plot shows the values of *R* vs. age: applying a linear fit, a significant negative trend was estimated (the 95% confidence interval for the slope was entirely negative [−0.00681, −0.00108]). Figure 1 shows that, as expected, gene expression is very similar between the two members of each twin couple (as represented by the very high value of correlation); however, a slight divergence occurs during ageing, possibly due to environmental causes or to an increase in “transcription noise”. We are not able to discriminate the causes of such divergence, but we remark that this observation could only be evaluated by an experimental design in which MZ twins are studied. Moreover, the plot suggests that the larger increase in divergence occurs mostly in old age (from 60–70 y onward) but the number of samples is probably too low to draw such a conclusion.

The heritability for human lifespan is believed to be about 25% ^22, 23^. Also, relatively high heritability estimates have been obtained for the expression of topmost active genes of old Danish twin couples^15^. However, no data are available to our knowledge on the divergence between old MZ twins regarding gene expression. Such divergence, however, is very little, suggesting that most of the changes of gene expression observed when comparing young and old MZ pairs are likely under genetic control.

### Selection of optimal age regression signature

**Figure 2:**
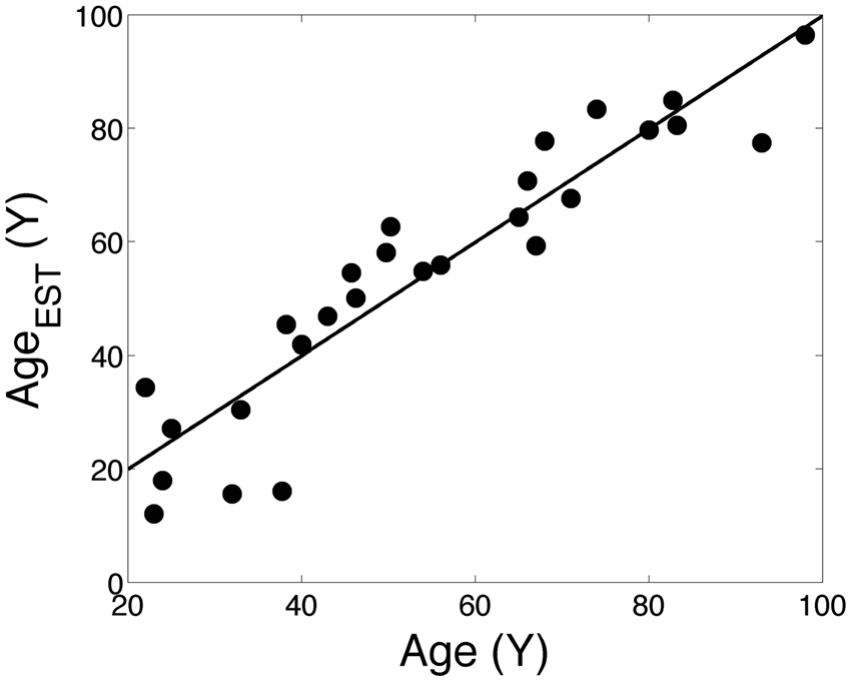
Plot of MZ couple age vs. age estimated by ridge regression with the optimal signature. The plot shows the estimation obtained for the validation set (i.e. all the twins from the 27 couples not used for signature training).

After this global observation, we considered the value of genetic divergence *Δ*_*i*_ for each single probe of the dataset (see Figure S1). Considering the z-score normalization of *Δ*_*i*_ (i.e. with 0 mean and unit standard deviation) we obtained a subset of probes with a z score <2.5 (about 300 probes, see Table S1). The regression procedure described in the Methods Section resulted in a 125-gene signature, shown in Table S2 (probes with an “unknown” annotation were removed from the signature). Pearson’s correlation analysis of regression output with samples’ age resulted in a value of R = 0.93 (results shown in Fig. 2 for the validation dataset obtained from the twin couple splitting, as described in Methods Section) to be compared with the results obtained in^21^ for a signature based on 71 methylation markers: R = 0.96 in the test dataset, and R = 0.91 in the validation set. As a first test for the goodness of our signature, we extracted from the Twins dataset 100,000 randomly chosen signatures of 125 probes, and applied the same procedure of estimating ridge regression parameters on one split dataset and validating it on the other. We obtained a distribution of 100,000 Pearson’s R values, with an average of 0.75 and a SD of 0.09. Remarkably, only 21 random signatures were outperforming the optimal signature (corresponding to 0.02% of the random signature sample space) confirming the high regression performance of the optimal age signature. Concerning possible gender differences in the expression values of these 125 genes, we did not observe any significant difference when comparing male to female twin samples, after correcting for multiple test analysis (data not shown). This suggests that such age signature is largely independent from sex differences, as evidenced also by the results for a dataset of male samples only (as shown in next section).

### Validation on a time series dataset of the same cell type

In order to test the robustness of our signature, we applied it to two different datasets. The first one (“Agegroup1” dataset, described in Remondini *et al*.^9^) consisted of T cells collected from peripheral blood of 25 male donors (age in the range 25–97 Y), analysed with the microarray platform HUMLIB384 Human OligoLibrary Release 1.0). The second dataset (“Agegroup2” dataset, GSE62331^24^), consisted of CD4+ T cells obtained from peripheral blood of 31 subjects (age in range 25–81 years; 17 males and 14 females), analysed with the microarray platform Illumina humanRef-8 v2.0.

Since different platforms have different probes, we considered the genes with the same “gene symbol” annotation as those of our signature. When more probes were associated with the same “gene symbol” we kept the one with the highest average expression value. This resulted in a reduced signature of 59 and 82 genes for the two datasets, respectively. The performance of these reduced signatures applied to the validation split dataset of MZ twins (with the same parameters of the 125-gene signature ridge regression) in terms of Pearson’s correlation coefficient, were R = 0.90 and R = 0.92, respectively.

**Figure 3:**
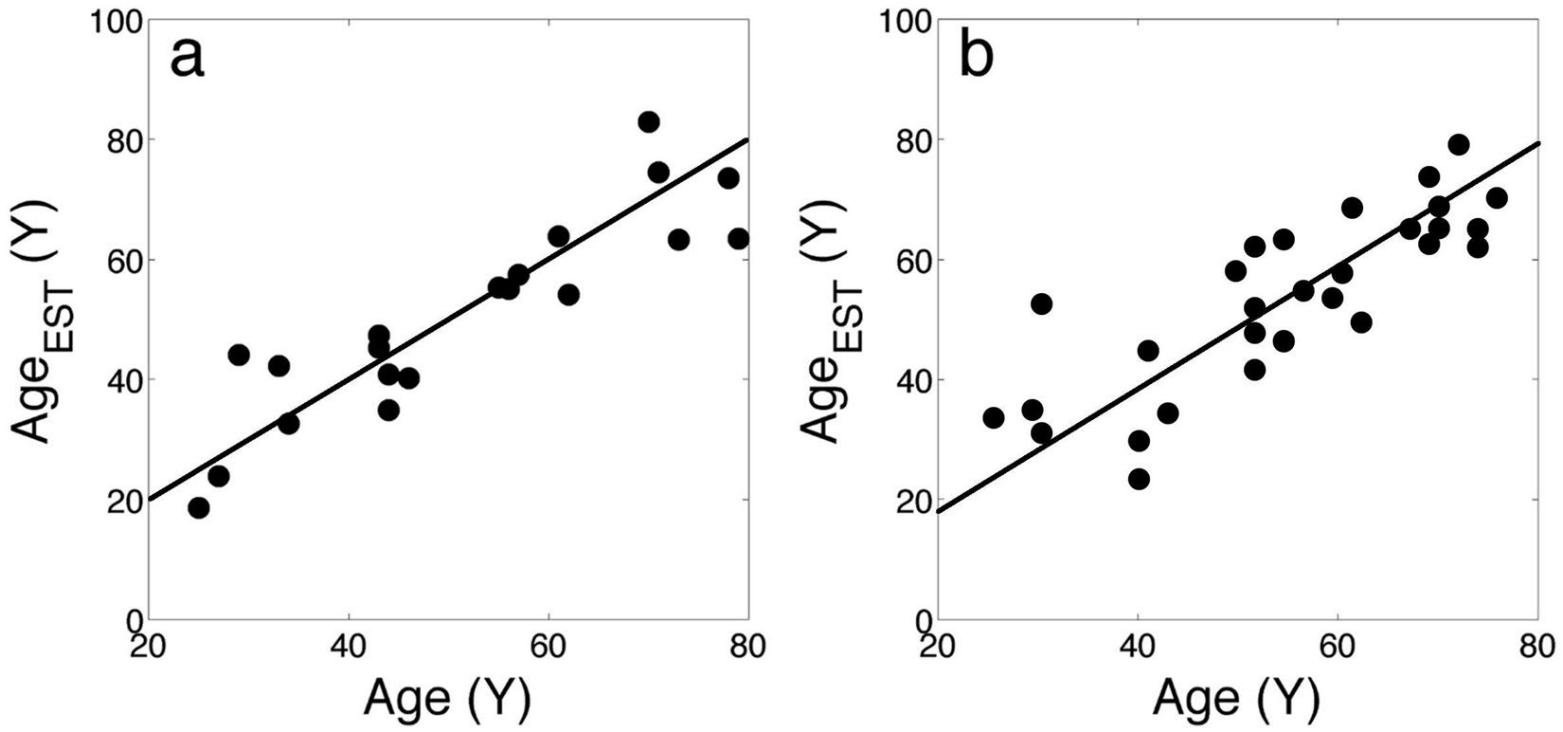
Plot of estimated age vs. real sample age for the Agegroup 1 (left) and the Agegroup 2 dataset (right). Pearson’s correlation coefficients in the two cases are R = 0.93 and R = 0.69, respectively.

In both datasets, a ridge regression was applied to the relative signature, in order to tune the signature to the different platform features. Applying the procedure without cross-validation, Pearson’s R = 0.95 and R = 0.99 were respectively obtained. More robust estimations were obtained by 10-fold crossvalidation, resampling 10,000 times the training and validation set: for the AgeGroup1 dataset, we obtained an average value R = 0.93 and a SD = 0.06 (see Fig. 3a), that is very similar to the performance obtained on the Twins dataset; for the Agegroup2 dataset, instead, we obtained an average value R = 0.69 and a SD = 0.04 (see Fig. 3b). Also for the two validation datasets, 10,000 randomly chosen signatures of appropriate length were extracted, a ridge regression procedure with 10-fold crossvalidation was applied, and the Pearson’s R was calculated for each random signature: in the AgeGroup1 dataset we obtained an average value R = 0.92 and a SD = 0.07, while in the Agegroup2 dataset we obtained an average value R = 0.41 and a SD = 0.14. In both cases, the distribution of the R values resulted lower for the random signature with respect to the reduced signature obtained from the Twins dataset. The average R value obtained using the Twins signature, in fact, was significantly higher than the random signatures in the Agegroup1 dataset (P = 10^−70^, Student’s T test, corresponding to the 60^th^ percentile of the distribution of R for random signatures) and to the 98^th^ percentile in the Agegroup2 dataset, suggesting that the lower average R obtained in the GSE62331 dataset is due to the data variability rather than to the signature.

Since the signature is applied on two datasets hybridized over two different microarray platforms with a reduced number of probes, the good performances obtained, as compared to random signatures, supports our approach for optimal signature selection based on the available twin experimental design.

### Validation on a time series of a different cell type

**Figure 4:**
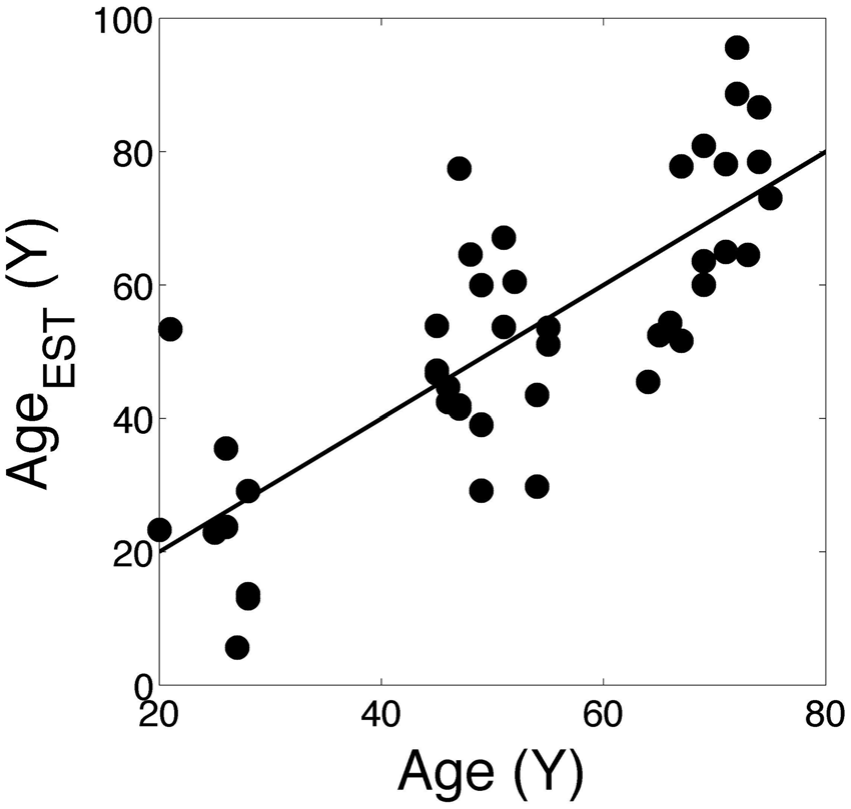
Plot of estimated age vs. real sample age for the Muscle dataset. Pearson’s correlation coefficient is R = 0.77.

We extracted, from GEO expression omnibus public repository for transcriptomic data, a dataset of skeletal muscle specimens extracted from healthy samples with ages ranging from 20 to 75 years, hybridized over the HG U133 Plus 2 microarray platform (“Muscle” dataset, GSE47881). The experimental design was a pre-post treatment (a resistance training performed by the subjects): we considered only the “pre” situation (supposed to be basal conditions before any treatment) resulting in a dataset of 44 samples. The genes found in this dataset with the same annotation than our signature were 83. With this reduced signature, we applied the ridge regression procedure 10,000 times with 10-fold cross-validation, obtaining an average Pearson’s R = 0.77, with a SD = 0.03 (see Fig. 4).

Generating 10,000 random signatures of the same dimensionality, analysed by 10-fold cross-validation as previously, we obtained an average performance with R = 0.64 and a SD = 0.11, and the original 83-gene signature corresponds to the 87th percentile of the distribution of the R obtained using a random signature.

In the case of the Muscle dataset, the signature performance higher than in Agegroup2 but not significantly higher than that of randomly chosen signatures. This result suggests a specificity of our signature for the T cells, but due to the uniqueness of the non T-cell dataset it is hard to draw a definitive conclusion.

### KEGG pathways analysis

In order to gain a deeper biological knowledge on the genes that result more stable over time in the twin couples, we performed a KEGG enrichment analysis on the set of genes with *Δ*_*i*_ smaller than the average by at least 1.5 standard deviations. In this way we obtained 420 significant probes (out of 3,978 probes in Codelink array with a known KEGG annotation, corresponding to 10.6% of the annotated probes, as listed in Table S3). KEGG analysis on these 420 probes (that include all the signature genes with KEGG annotation) identified 5 pathways significantly enriched (see Table 1) three of which are specific of T cell biology (“Antigen processing and presentation”; “T cell signalling”; “Leukocyte transendothelial migration”) thus enforcing the hypothesis of cell specificity of the age signature.

**Table 1:** List of significantly enriched KEGG pathways for the genes less varying in time with respect to twin couples (*Δ*_*i*_ < 1.5).

KeggId	Pathway Name	P-value	# Probes	# Significant	Ratio
4612	Antigen processing and presentation	0.0046	69	15	21.7391
4940	Type I diabetes mellitus	0.0059	39	10	25.641
4660	T cell receptor signaling pathway	0.0066	92	18	19.5652
4670	Leukocyte transendothelial migration	0.0110	111	20	18.018
4520	Adherens junction	0.0231	75	14	18.6667

KEGG Pathways.

## Discussion

Aging is a complex phenomenon during which a wide modification of gene expression has been reported for many organs and tissues in both humans and animal models^6, 9, 18, 25–30^. A plethora of studies have compared groups of individuals of different age in search for age-related changes of gene expression. As an example, in a recent meta-analysis of three large-scale transcriptomic studies, genes were grouped into networks of protein–protein interaction (PPI)^31^. Such a meta-analysis identified fifteen consistently coexpressed PPI modules associated with chronological age, including a highly significant module enriched for ‘T-cell activation’. Very recently a meta-analysis of studies of whole blood transcriptomics data identified 1,497 genes whose expression are differentially expressed with chronological age^19^. In this study 11,908 genes resulted significantly expressed across different platforms and were used to build a predictor for age. However, the studies on which these meta-analyses are built on considered unrelated singletons and, therefore, they identify genes whose changes likely reflect an adaptive response to individual clinical history and environmental living conditions. As a whole, the complexity of this topic and the general lack of concordance of the results obtained by the different studies, depending on many variables (ethnicity, type of analysed tissue, used platform, and, most importantly, the model of analysis) discouraged researchers from drawing definitive conclusions on “transcriptomics clocks”.

In order to offer a contribution in this topic, we propose to move the focus from genes that change their expression with age in the highest possible number of individuals (likely reflecting the average health status, the genetic background and the environmental living conditions of the considered population) to genes that change the less in genetically identical individuals, i.e. MZ twins, and that in addition change as a function of age. These genes should be those involved in normative aging (not driven by diseases or other incidental conditions). To test our hypothesis, we set up an approach based on ridge regression applied on a selection of genes. Our experimental design was based on monozygotic twin couples (male and female subjects) ranging from young adults to ultranonagenarians, thus we were able to define a score Δ in order to select those genes with minimal effects due to environmental genome variability. In fact, it has been demonstrated that there is a considerable proportion in the variation of gene expression that is likely attributable to genetic control and thus highly correlated in MZ twin pairs^15^.

A first interesting result of this study is that the concordance of the two members of the twin pairs decreases at advanced age, indicating that the subjects become progressively different from this point of view even if they are genetically identical. However, it is to note that such divergence, even if statistically significant, is very tiny, suggesting that still the great majority of the genes are expressed the same way in the members of the pair. This further suggests that the changes in expression characterizing normative aging are largely dictated by genetics and not by environmental factors.

Our procedure for probe selection and signature extraction led to an optimal age signature of 125 annotated genes. As expected, this signature is characterized by the presence of many genes involved in immune response, as highlighted by KEGG enrichment analysis, having been extracted from T lymphocytes purified from PBMC. We further verified the reliability of our signature by comparing the two top-ranking genes of the signature (i.e. with largest ridge coefficients), TRADD and HLA-DRB3, with data present in the literature. It is known that T lymphocytes express HLA-DR gene upon activation^32^, and that the percentage of circulating CD3 + HLA-DR+ T cells increases with age^33^. Accordingly, our data indicate for the probes specific for the gene of the beta chain of HLA-DR3 a strongly positive ridge coefficient. TNFRSF1A-associated via death domain (TRADD) is a death domain-containing adaptor molecule that interacts with TNFRSF1A/TNFR1 and mediates programmed cell death signaling and NF-κB activation. Its expression is reported to be higher in old subjects with respect to young ones^34^. In our dataset, the ridge coefficient of this gene is negative, indicating a trend of decreased expression with age; however, it is to note that in the study by Aggarwal *et al*. there was a simple comparison between two age groups and not a continuous age-range, as in our case. In fact the negative value of the ridge coefficient we got is due to the extremely high values of expression at middle age, which has not been considered in the study by Aggarwal *et al*. If we compare only young subjects (under 30 years) with those over-65 years, we observe that these latter have higher expression values than the former, in agreement with Aggarwal *et al*. It is to remark that we are considering a multidimensional signature, so the trend of a single gene isolated from the signature might not be as informative as if identified by single-gene analysis methods.

This signature was validated onto three different datasets, two of which performed on the same cell type but on a different hybridization platform. The signature, even with only a partial match between the annotation of the two platforms, resulted in a good performance, both in absolute value and as compared to a null hypothesis of random signatures of the same dimensionality. When the same signature was applied to a dataset differing both in terms of cell type and microarray platform, the performance was intermediate between the other two datasets (in terms of Pearson’s R) but not significantly higher as compared to a set of randomly chosen signatures of the same size. From these validation results we can thus conclude that our signature is highly performing, robust and platform independent, but most likely cell-type dependent, even if more dataset from different cell types should be analysed to reach a definitive conclusion regarding cell-type specificity. We tested also the level of overlapping between our signature and the more extended one identified by Peters *et al*.^19^. The intersection between the 125 genes of our signature and the 1,497 of Peters’ one identified only 11 shared genes: ‘EML4’, ‘PRPF19’, ‘SRSF1’, ‘EXOSC5’, ‘CHN1’, ‘GNG7’, ‘ZNF154’, ‘CHI3L2’, ‘RPL27A’, ‘CTSG’, ‘KLF12’. This indicates a very low level of overlapping between the two signatures and suggests that the changes in gene expression emerging in singletons are likely quite intense and overcome those identified with our method, except for these 11 genes. Further studies are needed to clarify the specific role of these genes in human aging. Furthermore, we wanted to check whether there is an overlap between our transcriptomic signature and epigenetic one identified by Horvath^20^. This signature, also indicated as “epigenetic clock” for its strong predictive power of chronological age, is based on the level of methylation of 353 CpG sites, and has the advantage of being not only very robust but also platform- and tissue-independent. In our signature only two genes (AKT3 and CHI3L2) out of 125 are in vicinity of CpGs present in Horvath’s signature. This lack of overlapping between the two signatures is somewhat expected, as the Horvath’s one is obtained from methylation data of 51 different tissues and cell types, while our own is from one single cell type.

An evident advantage for measuring a transcriptomic clock instead of an epigenetic one is that the gene expression levels should have a phenotypical correspondence in the cells/tissues, which is not always the case for DNA methylation, as it is known that age-associated changes in DNA methylation in general do not reflect those observed in gene expression^20^ with the exception of few genes^35^. However, the basic strength of our study relies in the experimental model (MZ twins with wide age range) and an analysis approach capable to fully exploit it, which allowed us to identify genes that were not noticed in previous studies and whose age-related expression changes are likely under genetic control. A limitation of our study is that, besides the limited number of twin pairs available for the analysis, it lacks functional parameters to be analysed in correlation of the expression data, so that we cannot support the idea that our signature works better than other proposed clocks in indicating the health status or the biological age of the studied subjects. Further studies are needed to clarify this point.

## Materials and Methods

### Experimental sample and expression microarrays

Gene expression data were obtained from resting T lymphocytes obtained from 15 female and 12 male homozygous twin couples, for a total of 54 subjects homogeneously distributed in an age span from 22 to 98 years (“Twin” dataset, GEO GSE74937, see Table S4). All the experiments were performed in accordance with the approved guidelines and regulations; the study and all the experimental protocols were approved by the Ethical Committee of the S. Orsola-Malpighi University Hospital (Bologna, Italy, ethical clearance #4/2002/U released on March 12, 2002). A signed informed consent was obtained from all participants. Peripheral blood mononuclear cells were obtained from 20 ml whole blood by Ficoll gradient separation. CD3^+^ T lymphocytes were separated by negative magnetic cell sorting (MACS, Miltenyi Biotec) using Pan T-cell Kit II (Miltenyi Biotec) microbeads. Purity and viability of samples were determined by flow cytometry, labelling T-cells respectively with mAb CD3-FITC and propidium iodide. Purity and viability were >95% in all cases. The resulting purified cell population was resuspended in TRIzol Reagent (Invitrogen, Paisley, UK) and stored at −80 °C before RNA extraction. CodeLink Human Whole Genome arrays (GE Healthcare) containing more than 55,000 probes were used to assess gene expression. Data were processed according to manufacturer’s instructions, and the samples were normalized using common procedures for such types of data: logarithmic transform and quantile normalization over the whole dataset.

### Extraction of gene expression signature

In order to exploit the information contained in the dataset about single-gene variability over time, we considered a score obtained from all the values for each gene in the microarray (indicated in the formula below with the symbol *g*_*i*_ for the i-th gene): we defined the measure *Δ*_*i*_ *(*eq. 1 *)*, the average absolute difference in expression between the twin couples (indicated by the apex numbers 1, 2 in the formula below) rescaled over the standard deviation (calculated from the expression profiles of both twins at all ages):

1 $${\Delta }_{i}=\frac{\langle |{g}_{i}^{1}-{g}_{i}^{2}|\rangle }{{\sigma }_{{g}_{i}}}$$

To define the optimal age signature, consisting in a low-dimensional subset of genes capable to recover the samples’ chronological age by regression methods, we applied a procedure organized into several steps. First, all the twin couples but one (thus by applying a leave-one-out cross-validation procedure) were used to calculate the mean absolute difference for each probe, and the probes with a value of *Δ*_*i*_ smaller than the average by at least 2.5 standard deviations were selected for the following step (see Figure S1 for a distribution of the score values without cross-validation). Secondly, the selected twin couples were randomly split into two datasets (one twin for each dataset), and a ridge regression (“ridge” function, Matlab software, with a ridge regression parameter *k* = 0.0001, see Supplementary Box) was performed on one half of the split dataset versus age, while the age of the other half of the dataset was estimated by this model for validation. Finally, the optimal gene signature was obtained by selecting the only genes appearing in all the 27 ridge regression estimated models.

To evaluate the goodness of the obtained signature, its performance has been compared with the performance of 100,000 signatures, with the same number of probes that have been randomly sampled from the whole twins dataset. Moreover, the same signature was applied to two different datasets (referred to as “Agegroup1” GSE99855, and “Agegroup2”, GSE62331) hybridized on different microarray platforms. The first consisted of 25 human male T cell samples of age ranging from 27 to 95 years (as described in Remondini *et al*.^9^) and the second consisted of CD4+ T cells obtained from peripheral blood of 31 subjects (age range 25–81 y; 17 males and 14 females). For the second dataset, batch effect was taken into account by including the replicate information in the regression, when available. The signature performance was compared against 10,000 randomly chosen signatures of the same size. We remark that since the microarray platforms have different annotations, only a subset of the probes from the original signature was considered for the analysis of the Agegroup datasets, resulting from the intersection of gene annotations in the Twins and the Agegroup platforms. The signature was further applied to another dataset with a similar time-series design but regarding a different cell type (muscle cells, GSE47881)^36^ and compared to 10,000 randomly chosen signatures of same dimensionality.

### Data Availability

The authors confirm that all relevant data are within the paper and its Supporting Information file.

## Electronic supplementary material

Supplementary Information
