# Supplementary material for: Identification of a T cell gene expression clock obtained by exploiting a MZ twin design
Source: Sci Rep. 2017 Jul 20;7:6005. doi: 10.1038/s41598-017-05694-2 (PMC5519672; doi:10.1038/s41598-017-05694-2)
Supplement: Supplementary file 1 — Supplementary Information [file 41598_2017_5694_MOESM1_ESM.docx]

# Identification of a T cell gene expression clock obtained by exploiting a MZ twin design

Daniel Remondini^1,2*^, Nathan Intrator^3*^, Claudia Sala^1^, Michela Pierini^4§^, Paolo Garagnani^2,4^, Isabella Zironi^1^, Claudio Franceschi^5^, Stefano Salvioli^2,4,#^, Gastone Castellani^1,2,#^

^1^Department of Physics and Astronomy, University of Bologna, Bologna 40126, Italy

^2^Interdepartmental Center "L. Galvani", University of Bologna, Bologna 40126, Italy

^3^Department of Computer Science, Exact Sciences Faculty, Tel Aviv University, Tel Aviv, Israel.

^4^Department of Experimental, Diagnostic and Specialty Medicine, University of Bologna, Bologna 40138, Italy

^5^IRCCS, Institute of Neurological Sciences of Bologna, Bologna 40124, Italy

*these authors equally contributed to the paper

^#^Senior co-authorship

^§^present address: Bone Regeneration Laboratory, Research Institute Codivilla-Putti, Rizzoli Orthopaedic Institute, Via di Barbiano 1/10, 40136 Bologna, Italy

**Supplementary material**

Supplementary Figure S1. Histogram of the Δ values for the 55,000 probes of the MZ twin dataset, after z-score normalization.


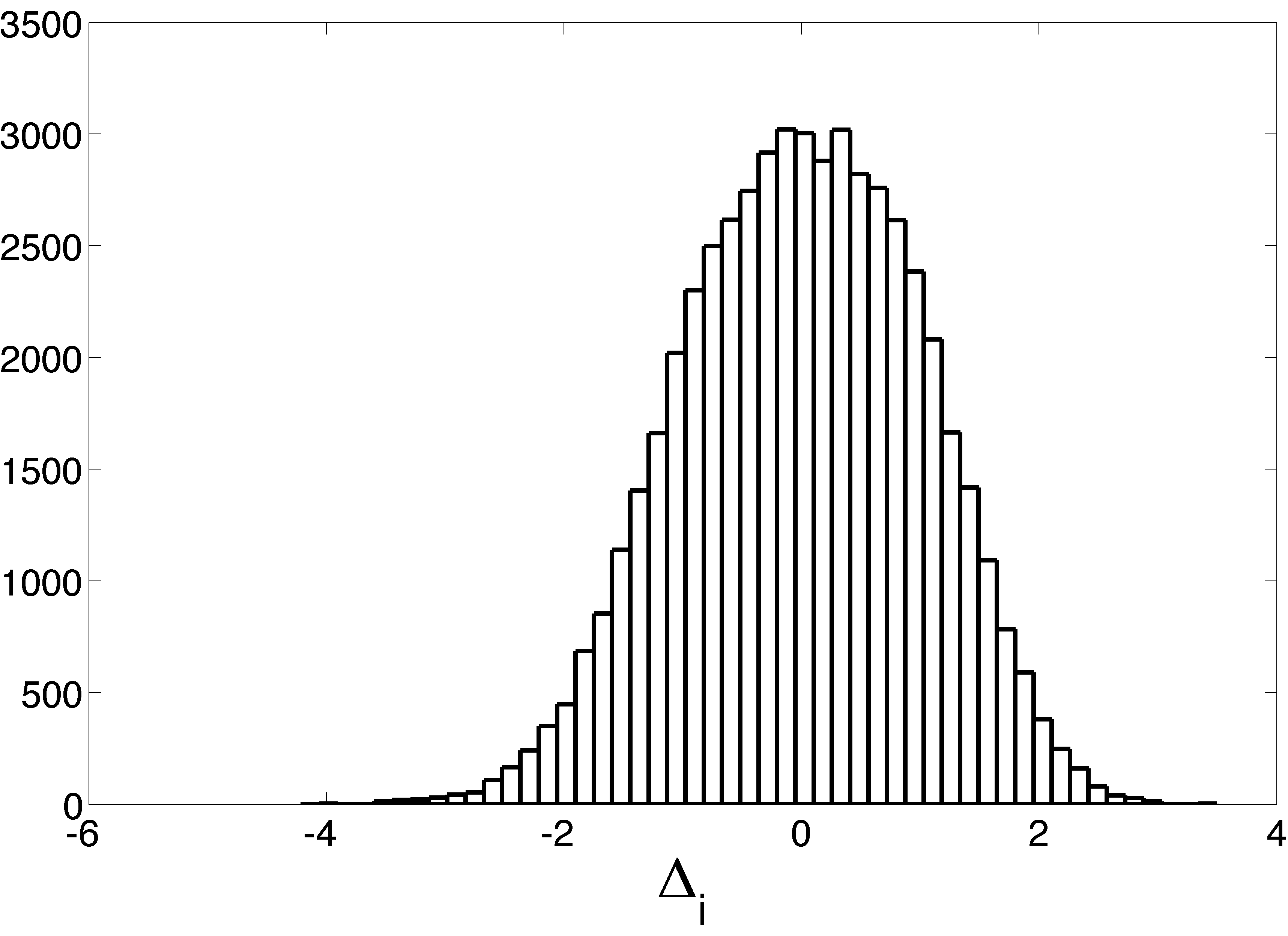


Supplementary Table S1. List of genes in MZ twin dataset with Δ<2.5σ_Δ_.

| **Probe** | **Gene** | **DiffVal** |
| --- | --- | --- |
| GE81687 | SDS | 0.0855168 |
| GE79014 | HLA-C | 0.116848 |
| GE672142 | NULL | 0.127152 |
| GE82055 | MRPL4 | 0.129923 |
| GE87316 | SEPT7 | 0.163943 |
| GE82058 | SDBCAG84 | 0.202625 |
| GE661797 | NULL | 0.20323 |
| GE59227 | SMCY | 0.203518 |
| GE82639 | SHFM3 | 0.210663 |
| GE59122 | SEMA3F | 0.211152 |
| GE83399 | CIP29 | 0.214232 |
| GE79837 | RPL27A | 0.216013 |
| GE58606 | DERP6 | 0.217547 |
| GE60019 | FAM12A | 0.223025 |
| GE845385 | NULL | 0.223425 |
| GE890404 | HLA-DQA1 | 0.224028 |
| GE589830 | NULL | 0.22526 |
| GE83443 | CYorf15B | 0.225436 |
| GE58177 | NULL | 0.226139 |
| GE578509 | FBXO13 | 0.227099 |
| GE522075 | NULL | 0.228219 |
| GE686106 | NULL | 0.229116 |
| GE81406 | MLLT3 | 0.231069 |
| GE54722 | SMP1 | 0.236384 |
| GE81181 | POLE2 | 0.237068 |
| GE87410 | NULL | 0.238285 |
| GE53749 | KIAA1107 | 0.238946 |
| GE83667 | MGC20255 | 0.239439 |
| GE571540 | NULL | 0.239666 |
| GE475883 | NULL | 0.239769 |
| GE79914 | NULL | 0.243561 |
| GE62702 | KCNJ4 | 0.2445 |
| GE847724 | NULL | 0.246949 |
| GE85269 | SLC11A2 | 0.247549 |
| GE53181 | KIAA0431 | 0.247641 |
| GE88455 | TREX1 | 0.251512 |
| GE624567 | KLF12 | 0.254174 |
| GE84148 | FLJ20203 | 0.254769 |
| GE493340 | NULL | 0.255859 |
| GE88082 | USP9Y | 0.258939 |
| GE87347 | FBXO9 | 0.259803 |
| GE79500 | RPS4Y | 0.261475 |
| GE789692 | NULL | 0.26243 |
| GE570933 | RPS4Y | 0.263444 |
| GE726206 | NULL | 0.265072 |
| GE829101 | NULL | 0.266386 |
| GE55146 | PSPH | 0.270922 |
| GE81179 | PLXNB1 | 0.272727 |
| GE59812 | RNASE3 | 0.272738 |
| GE78990 | LOC89894 | 0.273901 |
| GE88267 | SMAP-5 | 0.274068 |
| GE733799 | NULL | 0.274269 |
| GE80249 | NULL | 0.274568 |
| GE54029 | DDX3Y | 0.275174 |
| GE86679 | SFRS1 | 0.279629 |
| GE85842 | POLR2K | 0.280635 |
| GE540732 | NULL | 0.280904 |
| GE770922 | NULL | 0.281293 |
| GE834503 | NULL | 0.281801 |
| GE61737 | M17S2 | 0.284657 |
| GE80461 | C7orf34 | 0.285722 |
| GE62893 | LRAP | 0.28612 |
| GE88565 | HLA-DRB3 | 0.287052 |
| GE83222 | AGXT2L1 | 0.287617 |
| GE540515 | LOC284240 | 0.288883 |
| GE57860 | ANXA3 | 0.289046 |
| GE62837 | MAPK8IP1 | 0.28928 |
| GE88136 | NDUFA6 | 0.289527 |
| GE572395 | NULL | 0.290502 |
| GE646522 | HCLS1 | 0.291393 |
| GE86790 | TNC | 0.293089 |
| GE84306 | FLJ20635 | 0.293662 |
| GE472608 | SRA1 | 0.293725 |
| GE487008 | CACNG5 | 0.295176 |
| GE604744 | NULL | 0.297313 |
| GE58004 | HIST1H1D | 0.298026 |
| GE492469 | NULL | 0.29921 |
| GE775548 | NULL | 0.301164 |
| GE87295 | STIM2 | 0.303903 |
| GE582347 | NULL | 0.305031 |
| GE80703 | CX36 | 0.306238 |
| GE56729 | DKFZp434N2030 | 0.306759 |
| GE502488 | NULL | 0.307795 |
| GE476991 | MGC29784 | 0.308498 |
| GE84795 | NULL | 0.30912 |
| GE86910 | ATP8B4 | 0.309478 |
| GE88487 | NMP200 | 0.3098 |
| GE480647 | FLJ21777 | 0.31081 |
| GE86951 | MGC11242 | 0.311641 |
| GE80522 | TNFSF15 | 0.312388 |
| GE80288 | KIAA1228 | 0.31251 |
| GE527274 | NULL | 0.314023 |
| GE752953 | FLJ23790 | 0.316248 |
| GE556176 | NULL | 0.316389 |
| GE62998 | CEACAM3 | 0.31769 |
| GE58158 | CAPG | 0.31858 |
| GE878120 | NULL | 0.320827 |
| GE57347 | GML | 0.320937 |
| GE57894 | THRB | 0.322429 |
| GE842959 | NULL | 0.322467 |
| GE80810 | DKFZP564D0478 | 0.323156 |
| GE88657 | NALP5 | 0.323912 |
| GE79567 | MYL5 | 0.324374 |
| GE86519 | PSMB3 | 0.324693 |
| GE86149 | NULL | 0.325228 |
| GE81493 | HSPA1A | 0.32587 |
| GE87707 | MGC45400 | 0.326105 |
| GE57769 | TRADD | 0.327207 |
| GE539638 | NULL | 0.327506 |
| GE87602 | SCGB3A1 | 0.330039 |
| GE57486 | INHBA | 0.330183 |
| GE83840 | NULL | 0.330741 |
| GE544375 | ATP9A | 0.331083 |
| GE562325 | NULL | 0.331926 |
| GE63322 | FLJ14639 | 0.332361 |
| GE907010 | FLJ20573 | 0.332888 |
| GE61583 | VAMP8 | 0.333113 |
| GE57189 | PSME4 | 0.333143 |
| GE58849 | MAP2 | 0.333457 |
| GE691562 | NULL | 0.333546 |
| GE87456 | KIAA1797 | 0.334118 |
| GE511321 | NULL | 0.334285 |
| GE84953 | NULL | 0.335513 |
| GE62928 | CHI3L2 | 0.336144 |
| GE53152 | COX7A2L | 0.336443 |
| GE88420 | AKT3 | 0.338133 |
| GE475363 | ZNF154 | 0.338663 |
| GE88047 | FLJ31438 | 0.338799 |
| GE87317 | HTRA3 | 0.339275 |
| GE560971 | NULL | 0.339398 |
| GE53240 | FLJ00058 | 0.339625 |
| GE473297 | DEFT1 | 0.340269 |
| GE82400 | C11orf10 | 0.341271 |
| GE869099 | NULL | 0.34208 |
| GE631804 | NULL | 0.342323 |
| GE52942 | NULL | 0.343895 |
| GE58931 | TCF8 | 0.344177 |
| GE82229 | RHBDL2 | 0.344443 |
| GE62128 | RRP46 | 0.344764 |
| GE750142 | NULL | 0.345094 |
| GE63109 | GSTM4 | 0.346027 |
| GE540813 | NULL | 0.346664 |
| GE504575 | NULL | 0.34681 |
| GE86021 | MGC39350 | 0.347002 |
| GE722298 | NULL | 0.347585 |
| GE82245 | FLJ20619 | 0.347638 |
| GE825764 | FLJ31455 | 0.347726 |
| GE790852 | NULL | 0.347841 |
| GE59954 | MYOM2 | 0.348316 |
| GE84381 | NULL | 0.349076 |
| GE87840 | MGC27034 | 0.349158 |
| GE826802 | NULL | 0.34968 |
| GE86492 | SFRS3 | 0.34996 |
| GE84056 | ALDH3A2 | 0.350581 |
| GE473512 | NULL | 0.35067 |
| GE85202 | NULL | 0.351365 |
| GE59995 | DDR2 | 0.351525 |
| GE820932 | NULL | 0.352031 |
| GE561433 | NULL | 0.353177 |
| GE563052 | NULL | 0.354126 |
| GE59022 | PAK2 | 0.354662 |
| GE83775 | NULL | 0.35491 |
| GE62597 | FYCO1 | 0.355746 |
| GE81838 | UBQLN1 | 0.355937 |
| GE81271 | VIL2 | 0.356013 |
| GE834142 | NULL | 0.356949 |
| GE59753 | CHN1 | 0.356985 |
| GE81558 | CBF2 | 0.35728 |
| GE751776 | NULL | 0.35747 |
| GE793802 | NULL | 0.358604 |
| GE80157 | CYBA | 0.359122 |
| GE79166 | RETN | 0.359795 |
| GE57841 | CTSG | 0.359873 |
| GE59764 | MYL4 | 0.360429 |
| GE597795 | NULL | 0.360442 |
| GE774452 | NULL | 0.361248 |
| GE62231 | LRG | 0.362746 |
| GE855420 | NULL | 0.363192 |
| GE664098 | NULL | 0.363736 |
| GE85634 | ZNF496 | 0.364194 |
| GE526402 | LOC285248 | 0.364804 |
| GE81750 | ELL2 | 0.366013 |
| GE81894 | MKRN2 | 0.367065 |
| GE57091 | CREM | 0.367521 |
| GE56186 | MDGA1 | 0.368018 |
| GE57767 | COL9A3 | 0.3687 |
| GE55919 | EML4 | 0.368743 |
| GE850657 | NULL | 0.369071 |
| GE83247 | PNAS-131 | 0.369109 |
| GE86335 | NULL | 0.369568 |
| GE515649 | NULL | 0.369669 |
| GE86783 | LOC146174 | 0.369743 |
| GE549334 | NULL | 0.37015 |
| GE61584 | FXYD5 | 0.370491 |
| GE593055 | NULL | 0.37078 |
| GE60199 | CREBL1 | 0.370883 |
| GE696200 | NULL | 0.370967 |
| GE57001 | FLJ23584 | 0.371235 |
| GE63079 | FYN | 0.371311 |
| GE866204 | NULL | 0.372115 |
| GE475932 | SPACA1 | 0.372289 |
| GE61327 | HBB | 0.372673 |
| GE87998 | HIST1H4F | 0.372764 |
| GE79024 | FOS | 0.372949 |
| GE655708 | NULL | 0.373206 |
| GE563761 | FLJ25436 | 0.373795 |
| GE88833 | FANCC | 0.373849 |
| GE86336 | SLC38A1 | 0.374109 |
| GE87803 | TXL-2 | 0.374852 |
| GE54657 | DKFZP564D172 | 0.374984 |
| GE80231 | CPEB4 | 0.375045 |
| GE55734 | FLJ10404 | 0.375077 |
| GE505013 | LOC116143 | 0.375213 |
| GE81686 | COPS6 | 0.375673 |
| GE82952 | C6orf76 | 0.376129 |
| GE805669 | NULL | 0.376169 |
| GE57057 | GLO1 | 0.37618 |
| GE79383 | PGR1 | 0.376545 |
| GE80955 | FOLR3 | 0.376766 |
| GE82982 | C6orf155 | 0.376809 |
| GE54087 | GOSR2 | 0.376814 |
| GE791245 | NULL | 0.377154 |
| GE88726 | NULL | 0.378039 |
| GE86261 | MGC3036 | 0.378247 |
| GE88616 | SLU7 | 0.378703 |
| GE88852 | LCN2 | 0.37896 |
| GE88661 | C20orf36 | 0.379105 |
| GE87219 | LRP15 | 0.379278 |
| GE86955 | FLJ10769 | 0.379347 |
| GE59919 | DEFA4 | 0.379697 |
| GE62572 | VIPR1 | 0.38001 |
| GE81332 | PCAF | 0.381119 |
| GE87503 | AGS3 | 0.381656 |
| GE810553 | NULL | 0.381674 |
| GE54799 | EML2 | 0.381711 |
| GE81071 | CEBPE | 0.382547 |
| GE83769 | NKAP | 0.382711 |
| GE903125 | USP24 | 0.382959 |
| GE83905 | NULL | 0.383101 |
| GE83688 | SNX26 | 0.383106 |
| GE523582 | NULL | 0.383596 |
| GE58623 | CGI-18 | 0.383694 |
| GE62212 | FBLN2 | 0.384028 |
| GE58866 | TNFSF9 | 0.384654 |
| GE87762 | NULL | 0.384655 |
| GE642315 | NULL | 0.384661 |
| GE585042 | NULL | 0.384985 |
| GE61053 | FLJ23506 | 0.385084 |
| GE57856 | MPO | 0.385822 |
| GE87431 | FLJ23017 | 0.385844 |
| GE524164 | NULL | 0.385956 |
| GE81610 | PDE4D | 0.386016 |
| GE84523 | NULL | 0.38641 |
| GE88855 | GPIG4 | 0.386434 |
| GE652440 | NULL | 0.386633 |
| GE570443 | FLJ32844 | 0.387559 |
| GE53054 | NULL | 0.387691 |
| GE55382 | C10orf26 | 0.387907 |
| GE83877 | NULL | 0.388209 |
| GE81528 | LCN2 | 0.388848 |
| GE710949 | NULL | 0.388927 |
| GE714891 | NULL | 0.389334 |
| GE719445 | NULL | 0.38939 |
| GE84225 | NULL | 0.389688 |
| GE55731 | KIAA1576 | 0.389968 |
| GE53247 | ALEX2 | 0.390003 |
| GE59677 | S100A8 | 0.390242 |
| GE675960 | NULL | 0.390338 |
| GE57793 | PPP2R5E | 0.390349 |
| GE63000 | TERF1 | 0.390472 |
| GE81178 | PLGL | 0.391116 |
| GE61041 | ZNF174 | 0.391264 |
| GE831963 | NULL | 0.391848 |
| GE58122 | OSBP | 0.392082 |
| GE584844 | MDGA1 | 0.392167 |
| GE56086 | LOC90529 | 0.392255 |
| GE53666 | KLF3 | 0.392288 |
| GE61465 | KDELR2 | 0.392411 |
| GE86189 | FLJ22028 | 0.392624 |
| GE55911 | SBDS | 0.39273 |
| GE84699 | KCNJ15 | 0.392911 |
| GE717866 | LOC125150 | 0.393623 |
| GE79003 | ZNF331 | 0.393979 |
| GE62052 | NCF4 | 0.394178 |
| GE86640 | PAGE-5 | 0.394353 |
| GE82666 | FKSG14 | 0.395612 |
| GE85679 | LOC157697 | 0.395746 |
| GE547671 | SERPINB10 | 0.395816 |
| GE80976 | RPS26 | 0.39588 |
| GE687465 | NULL | 0.396025 |
| GE753779 | NULL | 0.39614 |
| GE537746 | FLJ21458 | 0.39652 |
| GE719174 | NULL | 0.396746 |
| GE82304 | MCM3APAS | 0.396951 |
| GE81552 | ARIH1 | 0.397133 |
| GE869526 | NULL | 0.397163 |
| GE83398 | NULL | 0.397201 |
| GE53952 | CSGlcA-T | 0.397415 |
| GE478803 | LOC91942 | 0.397629 |
| GE56352 | TIPARP | 0.397694 |
| GE584560 | NULL | 0.397783 |
| GE768175 | NULL | 0.397819 |
| GE541404 | LOC375264 | 0.398504 |
| GE62794 | NULL | 0.398976 |
| GE84275 | HLA-DRB3 | 0.39949 |
| GE85637 | HLA-DQA1 | 0.399552 |
| GE486046 | FLJ14712 | 0.400181 |
| GE80993 | CA6 | 0.400206 |
| GE86420 | UBE2H | 0.400318 |
| GE53927 | DKFZP434B0335 | 0.400511 |

Supplementary Table S2. Gene signature for MZ twin, Agegroup1, Agegroup2 and Muscle datasets.

| **MZ twins** | | | **Agegroup 1** | | **Agegroup 2** | | **Muscle** | |
| --- | --- | --- | --- | --- | --- | --- | --- | --- |
| **ProbeId** | **GeneName** | **RidgeCoef** | **ProbeId** | **Gene** | **ProbeId** | **Gene** | **ProbeId** | **Gene** |
| GE472608 | SRA1 | -1.4534 | U79271 | AKT3 | ILMN_10129 | SLC11A2 | 221008_s_at | AGXT2L1 |
| GE473297 | DEFT1 | 0.0174 | NM_000382 | ALDH3A2 | ILMN_15317 | ELL2 | 242879_x_at | AKT3 |
| GE475363 | ZNF154 | 0.4962 | NM_005139 | ANXA3 | ILMN_10890 | PSME4 | 210544_s_at | ALDH3A2 |
| GE476991 | MGC29784 | -0.9662 | AK025125 | ATP8B4 | ILMN_27933 | TRADD | 209369_at | ANXA3 |
| GE480647 | FLJ21777 | 1.303 | AB014511 | ATP9A | ILMN_19173 | ESYT2 | 240747_at | ATP8B4 |
| GE487008 | CACNG5 | 0.0372 | NM_014206 | C11orf10 | ILMN_14445 | PSPH | 216129_at | ATP9A |
| GE526402 | LOC285248 | 0.2535 | NM_001747 | CAPG | ILMN_9768 | UBQLN1 | 218213_s_at | C11orf10 |
| GE53152 | COX7A2L | -0.4287 | NM_001815 | CEACAM3 | ILMN_20882 | ATP8B4 | 231435_at | C7orf34 |
| GE53181 | KIAA0431 | -1.469 | U58514 | CHI3L2 | ILMN_18567 | FYCO1 | 221401_at | CACNG5 |
| GE53240 | FLJ00058 | -0.7256 | NM_001822 | CHN1 | ILMN_26574 | C12orf29 | 201850_at | CAPG |
| GE53749 | KIAA1107 | 1.0443 | NM_001853 | COL9A3 | ILMN_15769 | USP9Y | 210789_x_at | CEACAM3 |
| GE54029 | DDX3Y | 0.5143 | NM_004718 | COX7A2L | ILMN_6934 | EXOSC5 | 213060_s_at | CHI3L2 |
| GE540515 | LOC284240 | 0.5675 | NM_001911 | CTSG | ILMN_27377 | MAPK8IP1 | 212624_s_at | CHN1 |
| GE544375 | ATP9A | 1.1912 | AL049280 | CYorf15B | ILMN_2940 | EZR | 229069_at | CIP29 |
| GE54657 | DKFZP564D172 | -0.9966 | NM_006182 | DDR2 | ILMN_17542 | NFATC2IP | 204724_s_at | COL9A3 |
| GE54722 | SMP1 | 0.7003 | NM_004660 | DDX3Y | ILMN_23096 | SCGB3A1 | 201256_at | COX7A2L |
| GE55146 | PSPH | -0.6809 | NM_012081 | ELL2 | ILMN_5585 | SRSF1 | 241740_at | CREM |
| GE55919 | EML4 | 0.7242 | NM_019063 | EML4 | ILMN_25505 | POLR2K | 205653_at | CTSG |
| GE56186 | MDGA1 | 0.2373 | AK023397 | FYCO1 | ILMN_138451 | FAM91A1 | 229445_at | CYBA |
| GE56729 | DKFZp434N2030 | 0.0945 | NM_002066 | GML | ILMN_8579 | RPS4Y1 | 223646_s_at | CYorf15B |
| GE57091 | CREM | -0.0956 | NM_000850 | GSTM4 | ILMN_16223 | NBR1 | 235631_at | DDR2 |
| GE570933 | RPS4Y | 0.781 | NM_000519 | HBB | ILMN_29649 | SRSF3 | 205001_s_at | DDX3Y |
| GE57189 | PSME4 | 0.5524 | NM_005335 | HCLS1 | ILMN_22173 | ERAP2 | 244026_at | ELL2 |
| GE57347 | GML | -0.5431 | U50342 | HLA-C | ILMN_21416 | MYL5 | 233940_at | EML4 |
| GE57486 | INHBA | -0.0349 | AF085878 | HTRA3 | ILMN_2068 | TNFSF15 | 217568_at | FAM12A |
| GE57767 | COL9A3 | -0.1306 | NM_002192 | INHBA | ILMN_22105 | GNG7 | 238472_at | FBXO9 |
| GE57769 | TRADD | -1.6593 | NM_004981 | KCNJ4 | ILMN_22338 | SDS | 228316_at | FLJ31438 |
| GE57841 | CTSG | 0.2496 | AB029030 | KIAA1107 | ILMN_543 | ATMIN | 244145_at | FYCO1 |
| GE578509 | FBXO13 | 1.4912 | NM_017794 | KIAA1797 | ILMN_15073 | STIM2 | 208000_at | GML |
| GE57860 | ANXA3 | -0.4626 | AK022098 | KLF12 | ILMN_11651 | FBXW4 | 210912_x_at | GSTM4 |
| GE57894 | THRB | -0.3125 | AF191545 | LRAP | ILMN_29127 | FAM118A | 217232_x_at | HBB |
| GE58004 | HIST1H1D | 0.0501 | NM_005456 | MAPK8IP1 | ILMN_4563 | TMEM222 | 202957_at | HCLS1 |
| GE58158 | CAPG | -0.4445 | AF087987 | MDGA1 | ILMN_3985 | RETN | 214537_at | HIST1H1D |
| GE58606 | DERP6 | 0.2005 | NM_004529 | MLLT3 | ILMN_28804 | RPUSD3 | 237625_s_at | HLA-C |
| GE58849 | MAP2 | 0.962 | NM_015956 | MRPL4 | ILMN_29791 | KDM5D | 236203_at | HLA-DQA1 |
| GE58931 | TCF8 | -0.7256 | NM_002476 | MYL4 | ILMN_4003 | GSTM4 | 200800_s_at | HSPA1A |
| GE59022 | PAK2 | -1.1525 | NM_002477 | MYL5 | ILMN_23177 | SARNP | 228580_at | HTRA3 |
| GE59122 | SEMA3F | 0.8418 | NM_003970 | MYOM2 | ILMN_14720 | FOCAD | 210511_s_at | INHBA |
| GE59227 | SMCY | 1.0061 | NM_002490 | NDUFA6 | ILMN_5590 | MLLT3 | 211451_s_at | KCNJ4 |
| GE59753 | CHN1 | -1.0781 | NM_002673 | PLXNB1 | ILMN_1337 | FAM172A | 214098_at | KIAA1107 |
| GE59764 | MYL4 | -0.8341 | NM_002692 | POLE2 | ILMN_18198 | MDGA1 | 232875_at | KIAA1797 |
| GE59812 | RNASE3 | -0.0832 | NM_002795 | PSMB3 | ILMN_24514 | CXorf38 | 243089_at | KLF12 |
| GE59954 | MYOM2 | 0.6145 | D38521 | PSME4 | ILMN_20790 | ERGIC3 | 233474_at | LOC284240 |
| GE59995 | DDR2 | -1.2234 | NM_004577 | PSPH | ILMN_6945 | CYBA | 240338_at | LRAP |
| GE60019 | FAM12A | 0.8061 | NM_020415 | RETN | ILMN_9751 | MYOM2 | 241044_x_at | MAP2 |
| GE61327 | HBB | -0.9911 | NM_000990 | RPL27A | ILMN_19705 | POLE2 | 213014_at | MAPK8IP1 |
| GE61583 | VAMP8 | 0.8564 | AF086152 | SCGB3A1 | ILMN_20475 | CTSG | 242757_at | MDGA1 |
| GE61737 | M17S2 | -0.5778 | NM_004186 | SEMA3F | ILMN_23612 | TMEM116 | 204918_s_at | MLLT3 |
| GE62128 | RRP46 | 0.4923 | NM_006924 | SFRS1 | ILMN_17426 | CLHC1 | 223743_s_at | MRPL4 |
| GE62231 | LRG | -1.1681 | NM_003017 | SFRS3 | ILMN_6623 | HSPA1A | 217274_x_at | MYL4 |
| GE624567 | KLF12 | 0.9423 | AK024213 | SRA1 | ILMN_22628 | PLXNB1 | 205145_s_at | MYL5 |
| GE62597 | FYCO1 | -0.276 | AB040915 | STIM2 | ILMN_3178 | HLA-DRB5 | 243913_at | MYOM2 |
| GE62702 | KCNJ4 | -0.0103 | AK022264 | THRB | ILMN_20961 | PSMB3 | 202001_s_at | NDUFA6 |
| GE62837 | MAPK8IP1 | -0.5842 | X56160 | TNC | ILMN_2712 | CAPG | 244268_x_at | PAK2 |
| GE62893 | LRAP | 1.1658 | L41690 | TRADD | ILMN_15293 | ALDH3A2 | 215807_s_at | PLXNB1 |
| GE62928 | CHI3L2 | -0.4624 | NM_016381 | TREX1 | ILMN_8404 | COX7A2L | 205909_at | POLE2 |
| GE62998 | CEACAM3 | -0.331 | NM_013438 | UBQLN1 | ILMN_24488 | PRR15L | 202635_s_at | POLR2K |
| GE63109 | GSTM4 | 0.0848 | NM_003761 | VAMP8 | ILMN_21821 | EML4 | 201400_at | PSMB3 |
| GE63322 | FLJ14639 | -1.4966 | NM_003379 | VIL2 | ILMN_23736 | PAK2 | 237180_at | PSME4 |
| GE646522 | HCLS1 | 1.3021 |  |  | ILMN_138702 | THRB | 244819_x_at | PSPH |
| GE752953 | FLJ23790 | 0.5333 |  |  | ILMN_17791 | HIST1H1D | 220570_at | RETN |
| GE78990 | LOC89894 | 0.3034 |  |  | ILMN_29902 | CREM | 219489_s_at | RHBDL2 |
| GE79014 | HLA-C | -1.1467 |  |  | ILMN_10626 | PRPF19 | 206851_at | RNASE3 |
| GE79166 | RETN | -0.1336 |  |  | ILMN_23044 | LRG1 | 212044_s_at | RPL27A |
| GE79500 | RPS4Y | 0.7111 |  |  | ILMN_17144 | DDX3Y | 230378_at | SCGB3A1 |
| GE79567 | MYL5 | -0.1086 |  |  | ILMN_26090 | RAF1 | 205695_at | SDS |
| GE79837 | RPL27A | -0.762 |  |  | ILMN_139166 | RPL27A | 35666_at | SEMA3F |
| GE80157 | CYBA | 1.4231 |  |  | ILMN_29741 | NDUFA6 | 239384_at | SFRS1 |
| GE80288 | KIAA1228 | 0.2678 |  |  | ILMN_995 | EXOSC3 | 237485_at | SFRS3 |
| GE80461 | C7orf34 | 1.3753 |  |  | ILMN_10006 | ZNF496 | 237106_at | SLC11A2 |
| GE80522 | TNFSF15 | 0.5709 |  |  | ILMN_17021 | TMEM258 | 224864_at | SRA1 |
| GE80703 | CX36 | -0.3241 |  |  | ILMN_17606 | YIPF5 | 234140_s_at | STIM2 |
| GE80810 | DKFZP564D0478 | 0.8071 |  |  | ILMN_24401 | CCDC97 | 233130_at | THRB |
| GE81179 | PLXNB1 | -0.3915 |  |  | ILMN_10421 | CEBPZ | 243540_at | TNC |
| GE81181 | POLE2 | 0.6246 |  |  | ILMN_26790 | VAMP8 | 221085_at | TNFSF15 |
| GE81271 | VIL2 | -0.3823 |  |  | ILMN_12551 | TCEAL8 | 213443_at | TRADD |
| GE81406 | MLLT3 | 1.2669 |  |  | ILMN_10504 | HCLS1 | 34689_at | TREX1 |
| GE81493 | HSPA1A | -0.0964 |  |  | ILMN_28875 | HBB | 222991_s_at | UBQLN1 |
| GE81558 | CBF2 | 0.3076 |  |  | ILMN_25906 | TRMT10A | 228492_at | USP9Y |
| GE81687 | SDS | 0.7433 |  |  | ILMN_28657 | TMEM50A | 202546_at | VAMP8 |
| GE81750 | ELL2 | -0.9184 |  |  | ILMN_1036 | CHI3L2 | 238645_at | VIL2 |
| GE81838 | UBQLN1 | 0.3852 |  |  | ILMN_23822 | HLA-C | 217242_at | ZNF154 |
| GE82055 | MRPL4 | 0.9276 |  |  |  |  | 239411_at | ZNF496 |
| GE82058 | SDBCAG84 | 0.1047 |  |  |  |  |  |  |
| GE82229 | RHBDL2 | -0.6199 |  |  |  |  |  |  |
| GE82245 | FLJ20619 | 0.3012 |  |  |  |  |  |  |
| GE82400 | C11orf10 | -0.0379 |  |  |  |  |  |  |
| GE825764 | FLJ31455 | -2.4408 |  |  |  |  |  |  |
| GE82639 | SHFM3 | 1.0544 |  |  |  |  |  |  |
| GE83222 | AGXT2L1 | -0.225 |  |  |  |  |  |  |
| GE83399 | CIP29 | 0.0298 |  |  |  |  |  |  |
| GE83443 | CYorf15B | 0.6502 |  |  |  |  |  |  |
| GE83667 | MGC20255 | 0.652 |  |  |  |  |  |  |
| GE84056 | ALDH3A2 | -1.1102 |  |  |  |  |  |  |
| GE84148 | FLJ20203 | -0.8691 |  |  |  |  |  |  |
| GE84306 | FLJ20635 | 0.4698 |  |  |  |  |  |  |
| GE85269 | SLC11A2 | 1.0532 |  |  |  |  |  |  |
| GE85634 | ZNF496 | 0.8126 |  |  |  |  |  |  |
| GE85842 | POLR2K | 0.795 |  |  |  |  |  |  |
| GE86021 | MGC39350 | 0.3937 |  |  |  |  |  |  |
| GE86492 | SFRS3 | -0.8702 |  |  |  |  |  |  |
| GE86519 | PSMB3 | 0.0282 |  |  |  |  |  |  |
| GE86679 | SFRS1 | -1.3549 |  |  |  |  |  |  |
| GE86790 | TNC | 0.853 |  |  |  |  |  |  |
| GE86910 | ATP8B4 | 0.0956 |  |  |  |  |  |  |
| GE86951 | MGC11242 | 0.8929 |  |  |  |  |  |  |
| GE87295 | STIM2 | -0.3788 |  |  |  |  |  |  |
| GE87316 | SEPD7 | 0.0139 |  |  |  |  |  |  |
| GE87317 | HTRA3 | 0.4052 |  |  |  |  |  |  |
| GE87347 | FBXO9 | -0.2419 |  |  |  |  |  |  |
| GE87456 | KIAA1797 | -0.6647 |  |  |  |  |  |  |
| GE87602 | SCGB3A1 | 0.8305 |  |  |  |  |  |  |
| GE87707 | MGC45400 | 0.8351 |  |  |  |  |  |  |
| GE87840 | MGC27034 | 0.2593 |  |  |  |  |  |  |
| GE88047 | FLJ31438 | 1.4345 |  |  |  |  |  |  |
| GE88082 | USP9Y | 0.5291 |  |  |  |  |  |  |
| GE88136 | NDUFA6 | 0.3578 |  |  |  |  |  |  |
| GE88267 | SMAP-5 | 0.6524 |  |  |  |  |  |  |
| GE88420 | AKT3 | 0.3374 |  |  |  |  |  |  |
| GE88455 | TREX1 | 0.498 |  |  |  |  |  |  |
| GE88487 | NMP200 | 0.2469 |  |  |  |  |  |  |
| GE88565 | HLA-DRB3 | 1.8187 |  |  |  |  |  |  |
| GE88657 | NALP5 | 0.1203 |  |  |  |  |  |  |
| GE890404 | HLA-DQA1 | -0.7422 |  |  |  |  |  |  |
| GE907010 | FLJ20573 | -0.4512 |  |  |  |  |  |  |

Supplementary Table S3. List of genes in MZ twin dataset with Δ<1.5σ_Δ_.

| **ProbeId** | **Symbol** | **KEGG** |
| --- | --- | --- |
| GE626229 | ABL2 | 04012: ErbB signaling pathway |
| GE80315 | ABLIM2 | 04360: Axon guidance |
| GE80460 | ACACB | 00061: Fatty acid biosynthesis; 00620: Pyruvate metabolism; 00640: Propanoate metabolism; 04910: Insulin signaling pathway; 04920: Adipocytokine signaling pathway |
| GE58551 | ACAD9 | 00120: Bile acid biosynthesis; 00281: Geraniol degradation; 00624: 1- and 2-Methylnaphthalene degradation |
| GE60170 | ACOX2 | 03320: PPAR signaling pathway |
| GE81290 | ACOX3 | 00071: Fatty acid metabolism; 00592: alpha-Linolenic acid metabolism; 01040: Polyunsaturated fatty acid biosynthesis; 03320: PPAR signaling pathway |
| GE86749 | ACP1 | 00361: gamma-Hexachlorocyclohexane degradation; 00740: Riboflavin metabolism; 04520: Adherens junction |
| GE58259 | ACP5 | 00361: gamma-Hexachlorocyclohexane degradation; 00740: Riboflavin metabolism |
| GE84661 | ACTB | 01430: Cell Communication; 04510: Focal adhesion; 04520: Adherens junction; 04530: Tight junction; 04670: Leukocyte transendothelial migration; 04810: Regulation of actin cytoskeleton; 05130: Pathogenic Escherichia coli infection - EHEC; 05131: Pathogenic Escherichia coli infection - EPEC |
| GE80152 | ACTG1 | 01430: Cell Communication; 04510: Focal adhesion; 04520: Adherens junction; 04530: Tight junction; 04670: Leukocyte transendothelial migration; 04810: Regulation of actin cytoskeleton; 05110: Cholera - Infection; 05130: Pathogenic Escherichia coli infection - EHEC; 05131: Pathogenic Escherichia coli infection - EPEC |
| GE59645 | ADA | 00230: Purine metabolism |
| GE88276 | ADCY4 | 00230: Purine metabolism; 04020: Calcium signaling pathway; 04540: Gap junction; 04742: Taste transduction; 04912: GnRH signaling pathway; 04916: Melanogenesis |
| GE57103 | ADCYAP1R1 | 04080: Neuroactive ligand-receptor interaction |
| GE86660 | ADIPOR2 | 04920: Adipocytokine signaling pathway |
| GE58416 | ADK | 00230: Purine metabolism |
| GE59498 | AGL | 00500: Starch and sucrose metabolism |
| GE80348 | AGTRL1 | 04080: Neuroactive ligand-receptor interaction |
| GE58217 | AIRE | 04120: Ubiquitin mediated proteolysis |
| GE88420 | AKT3 | 04010: MAPK signaling pathway; 04012: ErbB signaling pathway; 04150: mTOR signaling pathway; 04210: Apoptosis; 04370: VEGF signaling pathway; 04510: Focal adhesion; 04530: Tight junction; 04620: Toll-like receptor signaling pathway; 04630: Jak-STAT signaling pathway; 04660: T cell receptor signaling pathway; 04662: B cell receptor signaling pathway; 04664: Fc epsilon RI signaling pathway; 04910: Insulin signaling pathway; 04920: Adipocytokine signaling pathway; 05210: Colorectal cancer; 05211: Renal cell carcinoma; 05212: Pancreatic cancer; 05213: Endometrial cancer; 05214: Glioma; 05215: Prostate cancer; 05218: Melanoma; 05220: Chronic myeloid leukemia; 05221: Acute myeloid leukemia; 05222: Small cell lung cancer; 05223: Non-small cell lung cancer |
| GE60231 | ALAS1 | 00260: Glycine, serine and threonine metabolism; 00860: Porphyrin and chlorophyll metabolism |
| GE60276 | ALCAM | 04514: Cell adhesion molecules (CAMs) |
| GE84056 | ALDH3A2 | 00010: Glycolysis / Gluconeogenesis; 00053: Ascorbate and aldarate metabolism; 00071: Fatty acid metabolism; 00120: Bile acid biosynthesis; 00220: Urea cycle and metabolism of amino groups; 00280: Valine, leucine and isoleucine degradation; 00310: Lysine degradation; 00340: Histidine metabolism; 00380: Tryptophan metabolism; 00410: beta-Alanine metabolism; 00561: Glycerolipid metabolism; 00620: Pyruvate metabolism; 00640: Propanoate metabolism; 00641: 3-Chloroacrylic acid degradation; 00650: Butanoate metabolism; 00903: Limonene and pinene degradation |
| GE58922 | ALDH3B1 | 00010: Glycolysis / Gluconeogenesis; 00340: Histidine metabolism; 00350: Tyrosine metabolism; 00360: Phenylalanine metabolism; 00980: Metabolism of xenobiotics by cytochrome P450 |
| GE80979 | ALDH5A1 | 00251: Glutamate metabolism; 00650: Butanoate metabolism |
| GE53172 | ALG1 | 00510: N-Glycan biosynthesis; 01030: Glycan structures - biosynthesis 1 |
| GE61564 | ALPL | 00361: gamma-Hexachlorocyclohexane degradation; 00790: Folate biosynthesis |
| GE59064 | AMHR2 | 04060: Cytokine-cytokine receptor interaction; 04350: TGF-beta signaling pathway |
| GE60534 | AMPD3 | 00230: Purine metabolism |
| GE87792 | ANAPC11 | 04110: Cell cycle; 04120: Ubiquitin mediated proteolysis |
| GE84462 | ANPEP | 00480: Glutathione metabolism; 04614: Renin-angiotensin system; 04640: Hematopoietic cell lineage |
| GE57800 | APBB1 | 05010: Alzheimer's disease |
| GE62938 | AREG | 04012: ErbB signaling pathway |
| GE833898 | ARHGEF2 | 05130: Pathogenic Escherichia coli infection - EHEC; 05131: Pathogenic Escherichia coli infection - EPEC |
| GE81547 | ARPC1B | 04810: Regulation of actin cytoskeleton |
| GE59209 | ARPC2 | 04810: Regulation of actin cytoskeleton |
| GE54069 | ARPC5 | 04810: Regulation of actin cytoskeleton; 05130: Pathogenic Escherichia coli infection - EHEC; 05131: Pathogenic Escherichia coli infection - EPEC |
| GE81052 | ASNS | 00252: Alanine and aspartate metabolism; 00910: Nitrogen metabolism |
| GE81083 | ATF2 | 04010: MAPK signaling pathway |
| GE84947 | ATP5J | 00190: Oxidative phosphorylation |
| GE84155 | ATP5O | 00190: Oxidative phosphorylation |
| GE57888 | ATP6V1B1 | 00190: Oxidative phosphorylation; 05120: Epithelial cell signaling in Helicobacter pylori infection |
| GE57734 | ATP6V1B2 | 00190: Oxidative phosphorylation; 05120: Epithelial cell signaling in Helicobacter pylori infection |
| GE497464 | B3GNT7 | 00533: Keratan sulfate biosynthesis; 01030: Glycan structures - biosynthesis 1 |
| GE62066 | B4GALT1 | 00052: Galactose metabolism; 00510: N-Glycan biosynthesis; 00533: Keratan sulfate biosynthesis; 00602: Glycosphingolipid biosynthesis - neo-lactoseries; 01030: Glycan structures - biosynthesis 1; 01031: Glycan structures - biosynthesis 2 |
| GE56131 | B4GALT5 | 00512: O-Glycan biosynthesis; 01030: Glycan structures - biosynthesis 1 |
| GE86114 | BCAT1 | 00280: Valine, leucine and isoleucine degradation; 00290: Valine, leucine and isoleucine biosynthesis; 00770: Pantothenate and CoA biosynthesis |
| GE79818 | BCL10 | 04660: T cell receptor signaling pathway; 04662: B cell receptor signaling pathway |
| GE54911 | BET1L | 04130: SNARE interactions in vesicular transport |
| GE60368 | BMPR1A | 04060: Cytokine-cytokine receptor interaction; 04350: TGF-beta signaling pathway |
| GE58163 | BRAF | 04010: MAPK signaling pathway; 04012: ErbB signaling pathway; 04150: mTOR signaling pathway; 04320: Dorso-ventral axis formation; 04510: Focal adhesion; 04650: Natural killer cell mediated cytotoxicity; 04720: Long-term potentiation; 04730: Long-term depression; 04810: Regulation of actin cytoskeleton; 04910: Insulin signaling pathway; 05210: Colorectal cancer; 05211: Renal cell carcinoma; 05212: Pancreatic cancer; 05213: Endometrial cancer; 05214: Glioma; 05215: Prostate cancer; 05216: Thyroid cancer; 05218: Melanoma; 05219: Bladder cancer; 05220: Chronic myeloid leukemia; 05221: Acute myeloid leukemia; 05223: Non-small cell lung cancer |
| GE57118 | BST1 | 00760: Nicotinate and nicotinamide metabolism; 04020: Calcium signaling pathway |
| GE58865 | BTD | 00780: Biotin metabolism |
| GE60490 | C2 | 04610: Complement and coagulation cascades |
| GE58106 | CA4 | 00910: Nitrogen metabolism |
| GE80993 | CA6 | 00910: Nitrogen metabolism |
| GE59931 | CA9 | 00910: Nitrogen metabolism |
| GE487008 | CACNG5 | 04010: MAPK signaling pathway |
| GE61253 | CACNG6 | 04010: MAPK signaling pathway |
| GE59001 | CASP6 | 01510: Neurodegenerative Diseases; 04210: Apoptosis; 05040: Huntington's disease |
| GE54287 | CAV2 | 04510: Focal adhesion |
| GE87401 | CBL | 04012: ErbB signaling pathway; 04120: Ubiquitin mediated proteolysis; 04630: Jak-STAT signaling pathway; 04660: T cell receptor signaling pathway; 04910: Insulin signaling pathway; 05220: Chronic myeloid leukemia |
| GE58142 | CCND3 | 04110: Cell cycle; 04115: p53 signaling pathway; 04310: Wnt signaling pathway; 04510: Focal adhesion; 04630: Jak-STAT signaling pathway |
| GE88019 | CCNE1 | 04110: Cell cycle; 04115: p53 signaling pathway; 05215: Prostate cancer; 05222: Small cell lung cancer |
| GE60033 | CCNG1 | 04115: p53 signaling pathway |
| GE553404 | CCR3 | 04060: Cytokine-cytokine receptor interaction |
| GE81076 | CCR7 | 04060: Cytokine-cytokine receptor interaction |
| GE79118 | CD24 | 04640: Hematopoietic cell lineage |
| GE58392 | CD28 | 04514: Cell adhesion molecules (CAMs); 04660: T cell receptor signaling pathway; 04940: Type I diabetes mellitus |
| GE58781 | CD34 | 04514: Cell adhesion molecules (CAMs); 04640: Hematopoietic cell lineage |
| GE60506 | CD3E | 04640: Hematopoietic cell lineage; 04660: T cell receptor signaling pathway |
| GE59674 | CD3G | 04640: Hematopoietic cell lineage; 04660: T cell receptor signaling pathway |
| GE80112 | CD7 | 04640: Hematopoietic cell lineage |
| GE61166 | CD99 | 04514: Cell adhesion molecules (CAMs); 04670: Leukocyte transendothelial migration |
| GE57709 | CDA | 00240: Pyrimidine metabolism |
| GE54494 | CDC23 | 04110: Cell cycle; 04120: Ubiquitin mediated proteolysis |
| GE86095 | CDC42 | 04010: MAPK signaling pathway; 04360: Axon guidance; 04370: VEGF signaling pathway; 04510: Focal adhesion; 04520: Adherens junction; 04530: Tight junction; 04660: T cell receptor signaling pathway; 04670: Leukocyte transendothelial migration; 04810: Regulation of actin cytoskeleton; 04912: GnRH signaling pathway; 05120: Epithelial cell signaling in Helicobacter pylori infection; 05130: Pathogenic Escherichia coli infection - EHEC; 05131: Pathogenic Escherichia coli infection - EPEC; 05211: Renal cell carcinoma; 05212: Pancreatic cancer |
| GE57828 | CDK4 | 04110: Cell cycle; 04115: p53 signaling pathway; 04530: Tight junction; 04660: T cell receptor signaling pathway; 05212: Pancreatic cancer; 05214: Glioma; 05218: Melanoma; 05219: Bladder cancer; 05220: Chronic myeloid leukemia; 05222: Small cell lung cancer; 05223: Non-small cell lung cancer |
| GE57557 | CDK5 | 04360: Axon guidance |
| GE59136 | CDKN2D | 04110: Cell cycle |
| GE60289 | CEBPA | 05221: Acute myeloid leukemia |
| GE79937 | CFL1 | 04360: Axon guidance; 04810: Regulation of actin cytoskeleton |
| GE79053 | CFL2 | 04360: Axon guidance; 04810: Regulation of actin cytoskeleton |
| GE61928 | CHIT1 | 00530: Aminosugars metabolism |
| GE81452 | CIR | 04330: Notch signaling pathway |
| GE57859 | CLTB | 05040: Huntington's disease |
| GE62915 | CMAS | 00530: Aminosugars metabolism |
| GE603934 | CNGA4 | 04740: Olfactory transduction |
| GE86395 | COL4A2 | 01430: Cell Communication; 04510: Focal adhesion; 04512: ECM-receptor interaction; 05222: Small cell lung cancer |
| GE80950 | COMT | 00350: Tyrosine metabolism |
| GE84728 | COX4I1 | 00190: Oxidative phosphorylation |
| GE81370 | COX5A | 00190: Oxidative phosphorylation |
| GE79831 | COX7B | 00190: Oxidative phosphorylation |
| GE56389 | CPT1B | 00071: Fatty acid metabolism; 03320: PPAR signaling pathway; 04920: Adipocytokine signaling pathway |
| GE55558 | CPT2 | 00071: Fatty acid metabolism; 03320: PPAR signaling pathway; 04920: Adipocytokine signaling pathway |
| GE62392 | CR2 | 04610: Complement and coagulation cascades; 04640: Hematopoietic cell lineage; 04662: B cell receptor signaling pathway |
| GE86992 | CREB3L2 | 04916: Melanogenesis; 05215: Prostate cancer |
| GE84971 | CREB5 | 05215: Prostate cancer |
| GE57335 | CRY1 | 04710: Circadian rhythm |
| GE57995 | CSF3R | 04060: Cytokine-cytokine receptor interaction; 04630: Jak-STAT signaling pathway; 04640: Hematopoietic cell lineage |
| GE53952 | CSGlcA-T | 00532: Chondroitin sulfate biosynthesis; 01030: Glycan structures - biosynthesis 1 |
| GE57745 | CSNK1A1 | 04310: Wnt signaling pathway; 04340: Hedgehog signaling pathway |
| GE59058 | CSNK1D | 04340: Hedgehog signaling pathway; 04540: Gap junction; 04710: Circadian rhythm |
| GE84513 | CSNK1E | 04310: Wnt signaling pathway; 04340: Hedgehog signaling pathway; 04710: Circadian rhythm |
| GE57970 | CSNK2A2 | 04310: Wnt signaling pathway; 04520: Adherens junction; 04530: Tight junction |
| GE538082 | CTBP2 | 04310: Wnt signaling pathway; 04330: Notch signaling pathway; 05220: Chronic myeloid leukemia |
| GE62276 | CTNNBIP1 | 04310: Wnt signaling pathway |
| GE62530 | CTPS | 00240: Pyrimidine metabolism |
| GE57841 | CTSG | 04080: Neuroactive ligand-receptor interaction; 04614: Renin-angiotensin system |
| GE86289 | CTSS | 04612: Antigen processing and presentation |
| GE484220 | CXCL16 | 04060: Cytokine-cytokine receptor interaction |
| GE80157 | CYBA | 04670: Leukocyte transendothelial migration |
| GE59686 | CYC1 | 00190: Oxidative phosphorylation |
| GE79813 | CYFIP2 | 04810: Regulation of actin cytoskeleton |
| GE62534 | CYP2J2 | 00590: Arachidonic acid metabolism; 00591: Linoleic acid metabolism |
| GE85996 | DAAM1 | 04310: Wnt signaling pathway |
| GE57275 | DGKD | 00561: Glycerolipid metabolism; 00564: Glycerophospholipid metabolism; 04070: Phosphatidylinositol signaling system |
| GE57071 | DHCR24 | 00100: Biosynthesis of steroids |
| GE53595 | DHDH | 00980: Metabolism of xenobiotics by cytochrome P450 |
| GE87686 | DHH | 04340: Hedgehog signaling pathway |
| GE80116 | DHRS1 | 00361: gamma-Hexachlorocyclohexane degradation; 00363: Bisphenol A degradation; 00624: 1- and 2-Methylnaphthalene degradation; 00626: Naphthalene and anthracene degradation; 00632: Benzoate degradation via CoA ligation; 00903: Limonene and pinene degradation |
| GE55941 | DHRS4 | 00590: Arachidonic acid metabolism |
| GE60491 | DLD | 00010: Glycolysis / Gluconeogenesis; 00020: Citrate cycle (TCA cycle); 00252: Alanine and aspartate metabolism; 00260: Glycine, serine and threonine metabolism; 00280: Valine, leucine and isoleucine degradation; 00620: Pyruvate metabolism |
| GE62200 | DNMT3A | 00271: Methionine metabolism |
| GE57616 | DRD4 | 04080: Neuroactive ligand-receptor interaction |
| GE714713 | DTX2 | 04330: Notch signaling pathway |
| GE57654 | DTYMK | 00240: Pyrimidine metabolism |
| GE61271 | DUSP1 | 04010: MAPK signaling pathway |
| GE53677 | DUSP10 | 04010: MAPK signaling pathway |
| GE690652 | DUSP4 | 04010: MAPK signaling pathway |
| GE768258 | DUSP7 | 04010: MAPK signaling pathway |
| GE574933 | E2F2 | 04110: Cell cycle; 05212: Pancreatic cancer; 05214: Glioma; 05215: Prostate cancer; 05218: Melanoma; 05219: Bladder cancer; 05220: Chronic myeloid leukemia; 05222: Small cell lung cancer; 05223: Non-small cell lung cancer |
| GE81017 | ECH1 | 00350: Tyrosine metabolism |
| GE57920 | EDG1 | 04080: Neuroactive ligand-receptor interaction |
| GE80361 | EDG3 | 04080: Neuroactive ligand-receptor interaction |
| GE55127 | EDG6 | 04080: Neuroactive ligand-receptor interaction |
| GE79894 | EFNA3 | 04360: Axon guidance |
| GE59035 | EFNA5 | 04360: Axon guidance |
| GE54107 | EI24 | 04115: p53 signaling pathway |
| GE62383 | ENDOG | 04210: Apoptosis |
| GE57617 | ENPEP | 04614: Renin-angiotensin system |
| GE86043 | EPB41 | 04530: Tight junction |
| GE57994 | EPHA2 | 04360: Axon guidance |
| GE60530 | FBP1 | 00010: Glycolysis / Gluconeogenesis; 00030: Pentose phosphate pathway; 00051: Fructose and mannose metabolism; 00710: Carbon fixation; 04910: Insulin signaling pathway |
| GE81762 | FBXO2 | 04120: Ubiquitin mediated proteolysis |
| GE60514 | FCGR1A | 04640: Hematopoietic cell lineage |
| GE84819 | FDFT1 | 00100: Biosynthesis of steroids; 00900: Terpenoid biosynthesis |
| GE82483 | FGF20 | 04010: MAPK signaling pathway; 04810: Regulation of actin cytoskeleton; 05218: Melanoma |
| GE82573 | FGF6 | 04010: MAPK signaling pathway; 04810: Regulation of actin cytoskeleton; 05218: Melanoma |
| GE53405 | FGF8 | 04010: MAPK signaling pathway; 04810: Regulation of actin cytoskeleton; 05218: Melanoma |
| GE61113 | FLNA | 04010: MAPK signaling pathway; 04510: Focal adhesion |
| GE61554 | FLOT2 | 04910: Insulin signaling pathway |
| GE85623 | FLT4 | 04060: Cytokine-cytokine receptor interaction |
| GE79024 | FOS | 04010: MAPK signaling pathway; 04620: Toll-like receptor signaling pathway; 04660: T cell receptor signaling pathway; 04662: B cell receptor signaling pathway; 05210: Colorectal cancer |
| GE88015 | FPRL1 | 04080: Neuroactive ligand-receptor interaction |
| GE54579 | FRAT2 | 04310: Wnt signaling pathway |
| GE53320 | FUT7 | 00602: Glycosphingolipid biosynthesis - neo-lactoseries; 01031: Glycan structures - biosynthesis 2 |
| GE60337 | FUT8 | 00510: N-Glycan biosynthesis; 00533: Keratan sulfate biosynthesis; 01030: Glycan structures - biosynthesis 1 |
| GE88531 | FYN | 04360: Axon guidance; 04510: Focal adhesion; 04520: Adherens junction; 04650: Natural killer cell mediated cytotoxicity; 04660: T cell receptor signaling pathway; 04664: Fc epsilon RI signaling pathway; 05130: Pathogenic Escherichia coli infection - EHEC; 05131: Pathogenic Escherichia coli infection - EPEC |
| GE80327 | GABARAPL1 | 04140: Regulation of autophagy |
| GE57684 | GADD45A | 04010: MAPK signaling pathway; 04110: Cell cycle; 04115: p53 signaling pathway |
| GE82030 | GADD45B | 04010: MAPK signaling pathway; 04110: Cell cycle; 04115: p53 signaling pathway |
| GE54484 | GJB3 | 01430: Cell Communication |
| GE57057 | GLO1 | 00620: Pyruvate metabolism |
| GE55671 | GMPPA | 00051: Fructose and mannose metabolism |
| GE61145 | GNAI3 | 04360: Axon guidance; 04530: Tight junction; 04540: Gap junction; 04670: Leukocyte transendothelial migration; 04730: Long-term depression; 04916: Melanogenesis |
| GE80066 | GNMT | 00260: Glycine, serine and threonine metabolism |
| GE54689 | GOSR1 | 04130: SNARE interactions in vesicular transport |
| GE54087 | GOSR2 | 04130: SNARE interactions in vesicular transport |
| GE83174 | GPR63 | 04080: Neuroactive ligand-receptor interaction |
| GE81443 | GRAP2 | 04660: T cell receptor signaling pathway |
| GE63004 | GRHPR | 00620: Pyruvate metabolism; 00630: Glyoxylate and dicarboxylate metabolism |
| GE79002 | GRM1 | 04020: Calcium signaling pathway; 04080: Neuroactive ligand-receptor interaction; 04540: Gap junction; 04720: Long-term potentiation; 04730: Long-term depression |
| GE61362 | GSN | 04810: Regulation of actin cytoskeleton |
| GE84610 | GSS | 00251: Glutamate metabolism; 00480: Glutathione metabolism |
| GE61688 | GSTM1 | 00480: Glutathione metabolism; 00980: Metabolism of xenobiotics by cytochrome P450 |
| GE63109 | GSTM4 | 00480: Glutathione metabolism; 00980: Metabolism of xenobiotics by cytochrome P450 |
| GE86108 | GSTT1 | 00480: Glutathione metabolism; 00980: Metabolism of xenobiotics by cytochrome P450 |
| GE59841 | GTF2B | 03022: Basal transcription factors |
| GE60388 | HAAO | 00380: Tryptophan metabolism |
| GE80899 | HADHA | 00062: Fatty acid elongation in mitochondria; 00071: Fatty acid metabolism; 00280: Valine, leucine and isoleucine degradation; 00310: Lysine degradation; 00380: Tryptophan metabolism; 00410: beta-Alanine metabolism; 00632: Benzoate degradation via CoA ligation; 00640: Propanoate metabolism; 00650: Butanoate metabolism; 00903: Limonene and pinene degradation; 00930: Caprolactam degradation; 01040: Polyunsaturated fatty acid biosynthesis |
| GE62366 | HAL | 00340: Histidine metabolism; 00910: Nitrogen metabolism |
| GE88491 | HARS2 | 00340: Histidine metabolism; 00970: Aminoacyl-tRNA biosynthesis |
| GE646522 | HCLS1 | 04530: Tight junction; 05130: Pathogenic Escherichia coli infection - EHEC; 05131: Pathogenic Escherichia coli infection - EPEC |
| GE54357 | HCRTR1 | 04080: Neuroactive ligand-receptor interaction |
| GE537019 | HES1 | 04330: Notch signaling pathway; 04950: Maturity onset diabetes of the young |
| GE59057 | HIF1A | 04150: mTOR signaling pathway; 05211: Renal cell carcinoma |
| GE60467 | HK1 | 00010: Glycolysis / Gluconeogenesis; 00051: Fructose and mannose metabolism; 00052: Galactose metabolism; 00500: Starch and sucrose metabolism; 00521: Streptomycin biosynthesis; 00530: Aminosugars metabolism |
| GE87081 | HK2 | 00010: Glycolysis / Gluconeogenesis; 00051: Fructose and mannose metabolism; 00052: Galactose metabolism; 00500: Starch and sucrose metabolism; 00521: Streptomycin biosynthesis; 00530: Aminosugars metabolism |
| GE79014 | HLA-C | 04514: Cell adhesion molecules (CAMs); 04612: Antigen processing and presentation; 04650: Natural killer cell mediated cytotoxicity; 04940: Type I diabetes mellitus |
| GE85654 | HLA-DPA1 | 04514: Cell adhesion molecules (CAMs); 04612: Antigen processing and presentation; 04940: Type I diabetes mellitus |
| GE81120 | HLA-DPB1 | 04514: Cell adhesion molecules (CAMs); 04612: Antigen processing and presentation; 04940: Type I diabetes mellitus |
| GE890404 | HLA-DQA1 | 04514: Cell adhesion molecules (CAMs); 04612: Antigen processing and presentation; 04940: Type I diabetes mellitus |
| GE88126 | HLA-DRA | 04514: Cell adhesion molecules (CAMs); 04612: Antigen processing and presentation; 04640: Hematopoietic cell lineage; 04940: Type I diabetes mellitus |
| GE88565 | HLA-DRB3 | 04514: Cell adhesion molecules (CAMs); 04612: Antigen processing and presentation; 04640: Hematopoietic cell lineage; 04940: Type I diabetes mellitus |
| GE84989 | HLA-F | 04514: Cell adhesion molecules (CAMs); 04612: Antigen processing and presentation; 04940: Type I diabetes mellitus |
| GE80237 | HLA-G | 04514: Cell adhesion molecules (CAMs); 04612: Antigen processing and presentation; 04650: Natural killer cell mediated cytotoxicity; 04940: Type I diabetes mellitus |
| GE81493 | HSPA1A | 04010: MAPK signaling pathway; 04612: Antigen processing and presentation |
| GE87585 | HSPA1B | 04010: MAPK signaling pathway; 04612: Antigen processing and presentation |
| GE81523 | HSPA1L | 04010: MAPK signaling pathway; 04612: Antigen processing and presentation |
| GE61748 | HSPA8 | 04010: MAPK signaling pathway; 04612: Antigen processing and presentation |
| GE847809 | ICA1 | 04940: Type I diabetes mellitus |
| GE80960 | ICAM2 | 04514: Cell adhesion molecules (CAMs); 04650: Natural killer cell mediated cytotoxicity |
| GE86461 | ID1 | 04350: TGF-beta signaling pathway |
| GE81127 | ID2 | 04350: TGF-beta signaling pathway |
| GE59744 | IDI1 | 00100: Biosynthesis of steroids; 00900: Terpenoid biosynthesis |
| GE86827 | IFNAR1 | 04060: Cytokine-cytokine receptor interaction; 04620: Toll-like receptor signaling pathway; 04630: Jak-STAT signaling pathway; 04650: Natural killer cell mediated cytotoxicity |
| GE53760 | IL23A | 04060: Cytokine-cytokine receptor interaction; 04630: Jak-STAT signaling pathway |
| GE80931 | IL8RA | 04060: Cytokine-cytokine receptor interaction; 05120: Epithelial cell signaling in Helicobacter pylori infection |
| GE57658 | IL8RB | 04060: Cytokine-cytokine receptor interaction; 05120: Epithelial cell signaling in Helicobacter pylori infection |
| GE85092 | ILVBL | 00290: Valine, leucine and isoleucine biosynthesis; 00650: Butanoate metabolism; 00660: C5-Branched dibasic acid metabolism; 00770: Pantothenate and CoA biosynthesis |
| GE58573 | IMPA2 | 00521: Streptomycin biosynthesis; 00562: Inositol phosphate metabolism; 04070: Phosphatidylinositol signaling system |
| GE839392 | IMPDH2 | 00230: Purine metabolism |
| GE57486 | INHBA | 04060: Cytokine-cytokine receptor interaction; 04350: TGF-beta signaling pathway |
| GE81330 | INPP4B | 00562: Inositol phosphate metabolism; 04070: Phosphatidylinositol signaling system |
| GE79726 | INSRR | 05215: Prostate cancer |
| GE85057 | IQGAP1 | 04520: Adherens junction; 04810: Regulation of actin cytoskeleton |
| GE58687 | IRAK4 | 04210: Apoptosis; 04620: Toll-like receptor signaling pathway |
| GE60425 | IRF3 | 04620: Toll-like receptor signaling pathway |
| GE59217 | IRF5 | 04620: Toll-like receptor signaling pathway |
| GE61911 | ITGB1 | 04360: Axon guidance; 04510: Focal adhesion; 04512: ECM-receptor interaction; 04514: Cell adhesion molecules (CAMs); 04670: Leukocyte transendothelial migration; 04810: Regulation of actin cytoskeleton; 05130: Pathogenic Escherichia coli infection - EHEC; 05131: Pathogenic Escherichia coli infection - EPEC; 05222: Small cell lung cancer |
| GE59824 | ITPKB | 00562: Inositol phosphate metabolism; 04020: Calcium signaling pathway; 04070: Phosphatidylinositol signaling system |
| GE58276 | IVD | 00280: Valine, leucine and isoleucine degradation |
| GE60040 | KHK | 00051: Fructose and mannose metabolism |
| GE85191 | KLK3 | 05215: Prostate cancer |
| GE79227 | LAMB1 | 01430: Cell Communication; 04510: Focal adhesion; 04512: ECM-receptor interaction; 05060: Prion disease; 05222: Small cell lung cancer |
| GE84802 | LAP3 | 00330: Arginine and proline metabolism |
| GE87788 | LEPR | 04060: Cytokine-cytokine receptor interaction; 04080: Neuroactive ligand-receptor interaction; 04630: Jak-STAT signaling pathway; 04920: Adipocytokine signaling pathway |
| GE58282 | LIF | 04060: Cytokine-cytokine receptor interaction; 04630: Jak-STAT signaling pathway |
| GE59690 | LIPC | 00561: Glycerolipid metabolism |
| GE85803 | LMNA | 01430: Cell Communication |
| GE58399 | MAD2L2 | 04110: Cell cycle |
| GE59997 | MAN1A1 | 00510: N-Glycan biosynthesis; 01030: Glycan structures - biosynthesis 1 |
| GE58443 | MAN1B1 | 00510: N-Glycan biosynthesis; 01030: Glycan structures - biosynthesis 1 |
| GE88131 | MAOA | 00220: Urea cycle and metabolism of amino groups; 00260: Glycine, serine and threonine metabolism; 00340: Histidine metabolism; 00350: Tyrosine metabolism; 00360: Phenylalanine metabolism; 00380: Tryptophan metabolism |
| GE80264 | MAP2K1IP1 | 04010: MAPK signaling pathway |
| GE81155 | MAP3K10 | 04010: MAPK signaling pathway |
| GE81619 | MAP3K12 | 04010: MAPK signaling pathway |
| GE79221 | MAPK3 | 04010: MAPK signaling pathway; 04012: ErbB signaling pathway; 04150: mTOR signaling pathway; 04320: Dorso-ventral axis formation; 04350: TGF-beta signaling pathway; 04360: Axon guidance; 04370: VEGF signaling pathway; 04510: Focal adhesion; 04520: Adherens junction; 04540: Gap junction; 04620: Toll-like receptor signaling pathway; 04650: Natural killer cell mediated cytotoxicity; 04664: Fc epsilon RI signaling pathway; 04720: Long-term potentiation; 04730: Long-term depression; 04810: Regulation of actin cytoskeleton; 04910: Insulin signaling pathway; 04912: GnRH signaling pathway; 04916: Melanogenesis; 04930: Type II diabetes mellitus; 05210: Colorectal cancer; 05211: Renal cell carcinoma; 05212: Pancreatic cancer; 05213: Endometrial cancer; 05214: Glioma; 05215: Prostate cancer; 05216: Thyroid cancer; 05218: Melanoma; 05219: Bladder cancer; 05220: Chronic myeloid leukemia; 05221: Acute myeloid leukemia; 05223: Non-small cell lung cancer |
| GE62837 | MAPK8IP1 | 04010: MAPK signaling pathway |
| GE60149 | MARS | 00271: Methionine metabolism; 00450: Selenoamino acid metabolism; 00970: Aminoacyl-tRNA biosynthesis |
| GE59996 | MCM4 | 04110: Cell cycle |
| GE57351 | MCM6 | 04110: Cell cycle |
| GE61277 | MDH2 | 00020: Citrate cycle (TCA cycle); 00620: Pyruvate metabolism; 00630: Glyoxylate and dicarboxylate metabolism; 00710: Carbon fixation; 00720: Reductive carboxylate cycle (CO2 fixation) |
| GE81151 | MEF2C | 04010: MAPK signaling pathway |
| GE54140 | MGAM | 00052: Galactose metabolism; 00500: Starch and sucrose metabolism |
| GE61942 | MGAT4A | 00510: N-Glycan biosynthesis; 01030: Glycan structures - biosynthesis 1 |
| GE80049 | MGST1 | 00480: Glutathione metabolism; 00980: Metabolism of xenobiotics by cytochrome P450 |
| GE85382 | MLLT4 | 04520: Adherens junction; 04530: Tight junction; 04670: Leukocyte transendothelial migration |
| GE57856 | MPO | 00360: Phenylalanine metabolism; 00680: Methane metabolism; 00940: Phenylpropanoid biosynthesis |
| GE58211 | MSH2 | 05210: Colorectal cancer |
| GE79525 | MSN | 04670: Leukocyte transendothelial migration; 04810: Regulation of actin cytoskeleton |
| GE55256 | MTMR1 | 00051: Fructose and mannose metabolism; 00530: Aminosugars metabolism; 00730: Thiamine metabolism; 00740: Riboflavin metabolism |
| GE88848 | MYL5 | 04510: Focal adhesion; 04530: Tight junction; 04670: Leukocyte transendothelial migration; 04810: Regulation of actin cytoskeleton |
| GE58484 | MYST4 | 00350: Tyrosine metabolism; 00360: Phenylalanine metabolism; 00564: Glycerophospholipid metabolism; 00624: 1- and 2-Methylnaphthalene degradation; 00632: Benzoate degradation via CoA ligation; 00903: Limonene and pinene degradation; 00960: Alkaloid biosynthesis II |
| GE59169 | NAGLU | 00531: Glycosaminoglycan degradation; 01032: Glycan structures - degradation |
| GE57968 | NCF1 | 04670: Leukocyte transendothelial migration |
| GE62052 | NCF4 | 04670: Leukocyte transendothelial migration |
| GE81306 | NCK2 | 04012: ErbB signaling pathway; 04360: Axon guidance; 04660: T cell receptor signaling pathway; 05130: Pathogenic Escherichia coli infection - EHEC; 05131: Pathogenic Escherichia coli infection - EPEC |
| GE81837 | NCKAP1 | 04810: Regulation of actin cytoskeleton |
| GE81501 | NCL | 05130: Pathogenic Escherichia coli infection - EHEC; 05131: Pathogenic Escherichia coli infection - EPEC |
| GE81621 | NCOR1 | 05040: Huntington's disease |
| GE79970 | NDUFA3 | 00190: Oxidative phosphorylation |
| GE88136 | NDUFA6 | 00190: Oxidative phosphorylation |
| GE56951 | NDUFV1 | 00190: Oxidative phosphorylation |
| GE85324 | NDUFV2 | 00190: Oxidative phosphorylation |
| GE79301 | NEFH | 01510: Neurodegenerative Diseases; 05030: Amyotrophic lateral sclerosis (ALS) |
| GE87715 | NEFL | 05030: Amyotrophic lateral sclerosis (ALS) |
| GE81665 | NEU3 | 00511: N-Glycan degradation; 00600: Sphingolipid metabolism; 01032: Glycan structures - degradation |
| GE61568 | NFATC3 | 04310: Wnt signaling pathway; 04360: Axon guidance; 04370: VEGF signaling pathway; 04650: Natural killer cell mediated cytotoxicity; 04660: T cell receptor signaling pathway; 04662: B cell receptor signaling pathway |
| GE82547 | NFKBIA | 04210: Apoptosis; 04620: Toll-like receptor signaling pathway; 04660: T cell receptor signaling pathway; 04662: B cell receptor signaling pathway; 04920: Adipocytokine signaling pathway; 05120: Epithelial cell signaling in Helicobacter pylori infection; 05215: Prostate cancer; 05220: Chronic myeloid leukemia; 05222: Small cell lung cancer |
| GE81165 | NFYA | 04612: Antigen processing and presentation |
| GE63345 | NFYC | 04612: Antigen processing and presentation |
| GE57836 | NGFR | 01510: Neurodegenerative Diseases; 04060: Cytokine-cytokine receptor interaction |
| GE62961 | NLGN3 | 04514: Cell adhesion molecules (CAMs) |
| GE473534 | NLN | 04614: Renin-angiotensin system |
| GE88035 | NMNAT1 | 00760: Nicotinate and nicotinamide metabolism |
| GE79600 | NOG | 04350: TGF-beta signaling pathway |
| GE82183 | NOTCH1 | 04320: Dorso-ventral axis formation; 04330: Notch signaling pathway |
| GE56584 | NOTCH2 | 04320: Dorso-ventral axis formation; 04330: Notch signaling pathway |
| GE59027 | NR3C1 | 04080: Neuroactive ligand-receptor interaction |
| GE79306 | NR4A2 | 01510: Neurodegenerative Diseases |
| GE87190 | NUDT2 | 00230: Purine metabolism; 00240: Pyrimidine metabolism |
| GE54976 | NUDT5 | 00230: Purine metabolism; 00500: Starch and sucrose metabolism; 00790: Folate biosynthesis |
| GE59733 | ODC1 | 00220: Urea cycle and metabolism of amino groups |
| GE83124 | OGT | 00512: O-Glycan biosynthesis; 01030: Glycan structures - biosynthesis 1 |
| GE59497 | P2RX4 | 04020: Calcium signaling pathway; 04080: Neuroactive ligand-receptor interaction |
| GE83431 | PARD6G | 04530: Tight junction |
| GE79679 | PARVG | 04510: Focal adhesion |
| GE58257 | PAX6 | 04950: Maturity onset diabetes of the young |
| GE81332 | PCAF | 04330: Notch signaling pathway |
| GE59716 | PCCA | 00280: Valine, leucine and isoleucine degradation; 00640: Propanoate metabolism |
| GE57671 | PDE4B | 00230: Purine metabolism |
| GE81610 | PDE4D | 00230: Purine metabolism |
| GE54175 | PDE6D | 00230: Purine metabolism |
| GE54533 | PDE8A | 00230: Purine metabolism |
| GE86300 | PDHA1 | 00010: Glycolysis / Gluconeogenesis; 00252: Alanine and aspartate metabolism; 00290: Valine, leucine and isoleucine biosynthesis; 00620: Pyruvate metabolism; 00650: Butanoate metabolism |
| GE86626 | PECAM1 | 04514: Cell adhesion molecules (CAMs); 04670: Leukocyte transendothelial migration |
| GE80904 | PGAM2 | 00010: Glycolysis / Gluconeogenesis |
| GE59614 | PGK1 | 00010: Glycolysis / Gluconeogenesis; 00710: Carbon fixation |
| GE62994 | PHKG2 | 04020: Calcium signaling pathway; 04910: Insulin signaling pathway |
| GE85962 | PIK3R1 | 04012: ErbB signaling pathway; 04070: Phosphatidylinositol signaling system; 04150: mTOR signaling pathway; 04210: Apoptosis; 04370: VEGF signaling pathway; 04510: Focal adhesion; 04620: Toll-like receptor signaling pathway; 04630: Jak-STAT signaling pathway; 04650: Natural killer cell mediated cytotoxicity; 04660: T cell receptor signaling pathway; 04662: B cell receptor signaling pathway; 04664: Fc epsilon RI signaling pathway; 04670: Leukocyte transendothelial migration; 04810: Regulation of actin cytoskeleton; 04910: Insulin signaling pathway; 04930: Type II diabetes mellitus; 05210: Colorectal cancer; 05211: Renal cell carcinoma; 05212: Pancreatic cancer; 05213: Endometrial cancer; 05214: Glioma; 05215: Prostate cancer; 05218: Melanoma; 05220: Chronic myeloid leukemia; 05221: Acute myeloid leukemia; 05222: Small cell lung cancer; 05223: Non-small cell lung cancer |
| GE79872 | PIK3R3 | 04012: ErbB signaling pathway; 04070: Phosphatidylinositol signaling system; 04150: mTOR signaling pathway; 04210: Apoptosis; 04370: VEGF signaling pathway; 04510: Focal adhesion; 04620: Toll-like receptor signaling pathway; 04630: Jak-STAT signaling pathway; 04650: Natural killer cell mediated cytotoxicity; 04660: T cell receptor signaling pathway; 04662: B cell receptor signaling pathway; 04664: Fc epsilon RI signaling pathway; 04670: Leukocyte transendothelial migration; 04810: Regulation of actin cytoskeleton; 04910: Insulin signaling pathway; 04930: Type II diabetes mellitus; 05210: Colorectal cancer; 05211: Renal cell carcinoma; 05212: Pancreatic cancer; 05213: Endometrial cancer; 05214: Glioma; 05215: Prostate cancer; 05218: Melanoma; 05220: Chronic myeloid leukemia; 05221: Acute myeloid leukemia; 05222: Small cell lung cancer; 05223: Non-small cell lung cancer |
| GE60262 | PIK3R4 | 04140: Regulation of autophagy |
| GE88032 | PLCG1 | 00562: Inositol phosphate metabolism; 04012: ErbB signaling pathway; 04020: Calcium signaling pathway; 04070: Phosphatidylinositol signaling system; 04370: VEGF signaling pathway; 04650: Natural killer cell mediated cytotoxicity; 04660: T cell receptor signaling pathway; 04664: Fc epsilon RI signaling pathway; 04670: Leukocyte transendothelial migration; 05110: Cholera - Infection; 05120: Epithelial cell signaling in Helicobacter pylori infection; 05214: Glioma; 05223: Non-small cell lung cancer |
| GE81179 | PLXNB1 | 04360: Axon guidance |
| GE61698 | PLXNC1 | 04360: Axon guidance |
| GE57443 | PMAIP1 | 04115: p53 signaling pathway |
| GE57796 | PMVK | 00100: Biosynthesis of steroids |
| GE79187 | PNLIPRP1 | 00561: Glycerolipid metabolism |
| GE57130 | POLD3 | 00230: Purine metabolism; 00240: Pyrimidine metabolism; 03030: DNA polymerase |
| GE81181 | POLE2 | 00230: Purine metabolism; 00240: Pyrimidine metabolism; 03030: DNA polymerase |
| GE62743 | POLI | 03030: DNA polymerase |
| GE55872 | POLR1B | 00230: Purine metabolism; 00240: Pyrimidine metabolism; 03020: RNA polymerase |
| GE57185 | POLR2E | 00230: Purine metabolism; 00240: Pyrimidine metabolism; 03020: RNA polymerase |
| GE85842 | POLR2K | 00230: Purine metabolism; 00240: Pyrimidine metabolism; 03020: RNA polymerase |
| GE61306 | POMC | 04916: Melanogenesis; 04920: Adipocytokine signaling pathway |
| GE55508 | PPA2 | 00190: Oxidative phosphorylation |
| GE58830 | PPM1A | 04010: MAPK signaling pathway |
| GE80255 | PPP1R3A | 04910: Insulin signaling pathway |
| GE62658 | PPP1R3B | 04910: Insulin signaling pathway |
| GE79502 | PPP2CB | 04310: Wnt signaling pathway; 04350: TGF-beta signaling pathway; 04530: Tight junction; 04730: Long-term depression |
| GE82091 | PRKAG2 | 04910: Insulin signaling pathway; 04920: Adipocytokine signaling pathway |
| GE87724 | PRKAR1A | 04210: Apoptosis; 04910: Insulin signaling pathway |
| GE79300 | PRKAR2A | 04210: Apoptosis; 04910: Insulin signaling pathway |
| GE88182 | PRKCQ | 04530: Tight junction; 04660: T cell receptor signaling pathway; 04920: Adipocytokine signaling pathway |
| GE61647 | PRKX | 04010: MAPK signaling pathway; 04020: Calcium signaling pathway; 04310: Wnt signaling pathway; 04340: Hedgehog signaling pathway; 04540: Gap junction; 04720: Long-term potentiation; 04740: Olfactory transduction; 04742: Taste transduction; 04910: Insulin signaling pathway; 04912: GnRH signaling pathway; 04916: Melanogenesis |
| GE80783 | PRKY | 04010: MAPK signaling pathway; 04020: Calcium signaling pathway; 04310: Wnt signaling pathway; 04340: Hedgehog signaling pathway; 04540: Gap junction; 04720: Long-term potentiation; 04740: Olfactory transduction; 04742: Taste transduction; 04910: Insulin signaling pathway; 04912: GnRH signaling pathway; 04916: Melanogenesis |
| GE61594 | PSMA3 | 03050: Proteasome |
| GE86846 | PSMA5 | 03050: Proteasome |
| GE86519 | PSMB3 | 03050: Proteasome |
| GE79699 | PSMB5 | 03050: Proteasome |
| GE507294 | PSMD12 | 03050: Proteasome |
| GE57041 | PTAFR | 04020: Calcium signaling pathway; 04080: Neuroactive ligand-receptor interaction |
| GE84415 | PTGDR | 04080: Neuroactive ligand-receptor interaction |
| GE62312 | PTGS2 | 00590: Arachidonic acid metabolism; 04370: VEGF signaling pathway; 05222: Small cell lung cancer |
| GE81349 | PTK2B | 04020: Calcium signaling pathway; 04650: Natural killer cell mediated cytotoxicity; 04670: Leukocyte transendothelial migration; 04912: GnRH signaling pathway |
| GE57927 | PTPN1 | 04520: Adherens junction; 04910: Insulin signaling pathway |
| GE59447 | PTPN11 | 04630: Jak-STAT signaling pathway; 04650: Natural killer cell mediated cytotoxicity; 04670: Leukocyte transendothelial migration; 04920: Adipocytokine signaling pathway; 05120: Epithelial cell signaling in Helicobacter pylori infection; 05211: Renal cell carcinoma; 05220: Chronic myeloid leukemia |
| GE86258 | PYCR1 | 00330: Arginine and proline metabolism |
| GE57832 | PYGL | 00500: Starch and sucrose metabolism; 04910: Insulin signaling pathway |
| GE60001 | RAB13 | 04530: Tight junction |
| GE79324 | RAB23 | 04340: Hedgehog signaling pathway |
| GE787622 | RAC1 | 04010: MAPK signaling pathway; 04310: Wnt signaling pathway; 04360: Axon guidance; 04370: VEGF signaling pathway; 04510: Focal adhesion; 04520: Adherens junction; 04620: Toll-like receptor signaling pathway; 04650: Natural killer cell mediated cytotoxicity; 04662: B cell receptor signaling pathway; 04664: Fc epsilon RI signaling pathway; 04670: Leukocyte transendothelial migration; 04810: Regulation of actin cytoskeleton; 05030: Amyotrophic lateral sclerosis (ALS); 05120: Epithelial cell signaling in Helicobacter pylori infection; 05210: Colorectal cancer; 05211: Renal cell carcinoma; 05212: Pancreatic cancer |
| GE79729 | RAD54B | 00500: Starch and sucrose metabolism; 00790: Folate biosynthesis |
| GE80269 | RASGRP2 | 04010: MAPK signaling pathway |
| GE81207 | RBL1 | 04110: Cell cycle; 04350: TGF-beta signaling pathway |
| GE61090 | RDH11 | 00051: Fructose and mannose metabolism; 00052: Galactose metabolism; 00120: Bile acid biosynthesis; 00260: Glycine, serine and threonine metabolism; 00363: Bisphenol A degradation; 00591: Linoleic acid metabolism; 00625: Tetrachloroethene degradation; 00650: Butanoate metabolism |
| GE81208 | RDX | 04810: Regulation of actin cytoskeleton |
| GE63316 | RERE | 05050: Dentatorubropallidoluysian atrophy (DRPLA) |
| GE54316 | RFNG | 04330: Notch signaling pathway |
| GE85894 | RPL14 | 03010: Ribosome |
| GE79837 | RPL27A | 03010: Ribosome |
| GE823635 | RPL30 | 03010: Ribosome |
| GE57529 | RPS23 | 03010: Ribosome |
| GE80976 | RPS26 | 03010: Ribosome |
| GE63017 | RPS28 | 03010: Ribosome |
| GE58003 | RPS6KB1 | 04012: ErbB signaling pathway; 04150: mTOR signaling pathway; 04350: TGF-beta signaling pathway; 04910: Insulin signaling pathway; 05221: Acute myeloid leukemia |
| GE61706 | RRAS | 04010: MAPK signaling pathway; 04530: Tight junction; 04810: Regulation of actin cytoskeleton |
| GE84782 | SDHB | 00020: Citrate cycle (TCA cycle); 00190: Oxidative phosphorylation |
| GE81687 | SDS | 00260: Glycine, serine and threonine metabolism; 00272: Cysteine metabolism |
| GE59122 | SEMA3F | 04360: Axon guidance |
| GE893450 | SESN3 | 04115: p53 signaling pathway |
| GE81225 | SIAH1 | 04115: p53 signaling pathway; 04120: Ubiquitin mediated proteolysis; 04310: Wnt signaling pathway |
| GE80987 | SLC25A4 | 04020: Calcium signaling pathway |
| GE56944 | SMURF1 | 04120: Ubiquitin mediated proteolysis; 04350: TGF-beta signaling pathway |
| GE79259 | SNAP29 | 04130: SNARE interactions in vesicular transport |
| GE57476 | SRF | 04010: MAPK signaling pathway |
| GE59679 | SRPR | 03060: Protein export |
| GE86072 | STK4 | 04010: MAPK signaling pathway; 05223: Non-small cell lung cancer |
| GE58311 | STX16 | 04130: SNARE interactions in vesicular transport |
| GE62747 | SUCLA2 | 00020: Citrate cycle (TCA cycle); 00640: Propanoate metabolism; 00660: C5-Branched dibasic acid metabolism; 00720: Reductive carboxylate cycle (CO2 fixation) |
| GE83749 | SUFU | 04340: Hedgehog signaling pathway; 05217: Basal cell carcinoma |
| GE56813 | SYNJ2 | 00562: Inositol phosphate metabolism; 04070: Phosphatidylinositol signaling system |
| GE79129 | TAF1 | 03022: Basal transcription factors |
| GE82349 | TAF6L | 03022: Basal transcription factors |
| GE80097 | TAF9 | 03022: Basal transcription factors |
| GE80060 | TAS2R14 | 04742: Taste transduction |
| GE738883 | TFG | 05216: Thyroid cancer |
| GE57894 | THRB | 04080: Neuroactive ligand-receptor interaction |
| GE557954 | TLN2 | 04510: Focal adhesion |
| GE56281 | TLR1 | 04620: Toll-like receptor signaling pathway |
| GE85542 | TMSB4X | 04810: Regulation of actin cytoskeleton |
| GE86790 | TNC | 01430: Cell Communication; 04510: Focal adhesion; 04512: ECM-receptor interaction |
| GE54131 | TNFRSF10B | 04060: Cytokine-cytokine receptor interaction; 04115: p53 signaling pathway; 04210: Apoptosis; 04650: Natural killer cell mediated cytotoxicity |
| GE81929 | TNFRSF21 | 04060: Cytokine-cytokine receptor interaction |
| GE591251 | TNFSF8 | 04060: Cytokine-cytokine receptor interaction |
| GE58866 | TNFSF9 | 04060: Cytokine-cytokine receptor interaction |
| GE81259 | TNR | 01430: Cell Communication; 04510: Focal adhesion; 04512: ECM-receptor interaction |
| GE57769 | TRADD | 04210: Apoptosis; 04920: Adipocytokine signaling pathway |
| GE57147 | TRIP12 | 04120: Ubiquitin mediated proteolysis |
| GE902289 | TRPV1 | 04080: Neuroactive ligand-receptor interaction |
| GE79236 | TXK | 04670: Leukocyte transendothelial migration |
| GE59386 | UBE2C | 04120: Ubiquitin mediated proteolysis |
| GE61672 | UBE2D2 | 04120: Ubiquitin mediated proteolysis |
| GE79686 | UBE2D3 | 04120: Ubiquitin mediated proteolysis |
| GE86420 | UBE2H | 04120: Ubiquitin mediated proteolysis |
| GE86409 | UBE2I | 04120: Ubiquitin mediated proteolysis |
| GE62989 | UBE2J2 | 04120: Ubiquitin mediated proteolysis; 05020: Parkinson's disease |
| GE55131 | UBE2L3 | 04120: Ubiquitin mediated proteolysis; 05020: Parkinson's disease |
| GE85923 | UBE2N | 04120: Ubiquitin mediated proteolysis |
| GE55486 | UBE2R2 | 04120: Ubiquitin mediated proteolysis |
| GE83130 | ULBP1 | 04650: Natural killer cell mediated cytotoxicity |
| GE472825 | ULBP2 | 04650: Natural killer cell mediated cytotoxicity |
| GE57485 | UMPS | 00240: Pyrimidine metabolism |
| GE86494 | UQCRC2 | 00190: Oxidative phosphorylation |
| GE54801 | UROD | 00860: Porphyrin and chlorophyll metabolism |
| GE62982 | VAMP2 | 04130: SNARE interactions in vesicular transport |
| GE61583 | VAMP8 | 04130: SNARE interactions in vesicular transport |
| GE84830 | VDAC2 | 04020: Calcium signaling pathway |
| GE62572 | VIPR1 | 04080: Neuroactive ligand-receptor interaction |
| GE56100 | VNN2 | 00930: Caprolactam degradation |
| GE80697 | VNN3 | 00930: Caprolactam degradation |
| GE87873 | WARS2 | 00380: Tryptophan metabolism; 00970: Aminoacyl-tRNA biosynthesis |
| GE88130 | WAS | 04520: Adherens junction; 04810: Regulation of actin cytoskeleton; 05130: Pathogenic Escherichia coli infection - EHEC; 05131: Pathogenic Escherichia coli infection - EPEC |
| GE85798 | WASF2 | 04520: Adherens junction; 04810: Regulation of actin cytoskeleton |
| GE57430 | WASL | 04520: Adherens junction; 04810: Regulation of actin cytoskeleton; 05130: Pathogenic Escherichia coli infection - EHEC; 05131: Pathogenic Escherichia coli infection - EPEC |
| GE79116 | WWP1 | 04120: Ubiquitin mediated proteolysis; 05050: Dentatorubropallidoluysian atrophy (DRPLA) |
| GE63068 | YWHAH | 04110: Cell cycle |
| GE53044 | ZFYVE16 | 04350: TGF-beta signaling pathway |
| GE80296 | ZNRD1 | 00230: Purine metabolism; 00240: Pyrimidine metabolism; 03020: RNA polymerase |

Supplementary Table S4. Description of MZ twin samples (Age, Sex).

| **Array ID** | **Sex** | **Age** |
| --- | --- | --- |
| T00298696 | F | 22 |
| T00298697 | F | 22 |
| T00298668 | F | 23 |
| T00298669 | F | 23 |
| T00298700 | M | 24 |
| T00298701 | M | 24 |
| T00298707 | M | 25 |
| T00298708 | M | 25 |
| T00298698 | M | 32 |
| T00298699 | M | 32 |
| T00298674 | M | 33 |
| T00298675 | M | 33 |
| T00298504 | F | 38 |
| T00298505 | F | 38 |
| T00298666 | F | 38 |
| T00298667 | F | 38 |
| T00298709 | M | 40 |
| T00298710 | M | 40 |
| T00298662 | F | 43 |
| T00298663 | F | 43 |
| T00298308 | M | 46 |
| T00298468 | M | 46 |
| T00298644 | F | 46 |
| T00298645 | F | 46 |
| T00298664 | F | 50 |
| T00298665 | F | 50 |
| T00298705 | M | 50 |
| T00298706 | M | 50 |
| T00298647 | F | 54 |
| T00298648 | F | 54 |
| T00298469 | M | 56 |
| T00298470 | M | 56 |
| T00298462 | M | 65 |
| T00298464 | M | 65 |
| T00298676 | F | 66 |
| T00298678 | F | 66 |
| T00298670 | F | 67 |
| T00298671 | F | 67 |
| T00298711 | M | 68 |
| T00298712 | M | 68 |
| T00298309 | F | 71 |
| T00298680 | F | 71 |
| T00298472 | M | 74 |
| T00298681 | M | 74 |
| T00298658 | F | 80 |
| T00298661 | F | 80 |
| T00298465 | M | 83 |
| T00298466 | M | 83 |
| T00298672 | F | 83 |
| T00298673 | F | 83 |
| T00298601 | F | 93 |
| T00298602 | F | 93 |
| T00298306 | F | 98 |
| T00298307 | F | 98 |

Supplementary Box: ridge regression method

| Ridge regression is a generalization of the problem of multivariate linear regression of a variable y against a vector of values $\vec{x}$, in which the problem of square error minimization  $\begin{matrix} \text{min}\left( \left\Vert y-\vec{a}\cdot\vec{x}' \right\Vert^{2} \right) & \left( 1s \right) \end{matrix}$  is substituted by the following:  $\begin{matrix} \text{min}\left( \left\Vert y-\vec{a}\cdot\vec{x}' \right\Vert^{2}\text{+λ}\left\Vert\vec{a} \right\Vert^{2} \right) & \left( 2s \right) \end{matrix}$  From a mathematical point of view, since the solution of problem (1s) requires a matrix inversion, problem (2s) introduces an additional term (also known as Tichonov regularization) that avoids matrix singularities or bad conditioning. From a statistical point of view, it can be shown that this term introduces a “regularization” on the regression algorithm, so to reduce the weight of low-variance variables on the fit. |
| --- |
